# CNOT7 facilitates radiation resistance in colorectal cancer through TRIM21/XRCC6-mediated non-homologous end joining repair

**DOI:** 10.1038/s41419-025-08160-4

**Published:** 2025-11-17

**Authors:** Yien Li, Luying Cui, Shaoke Wang, Qunye Zhao, Fenqi Du, Songtao Du, Chenfeng Yu, Mingyu Xia, Shihui Zhao, Tian Luan, Yanlong Liu, Binbin Cui

**Affiliations:** 1 Department of Colorectal Surgery, Harbin Medical University Cancer Hospital, Harbin, China; 2 Department of Gastrointestinal Medical Oncology, Harbin Medical University Cancer Hospital, Harbin, China; 3 Key Laboratory of Tumor Immunology in Heilongjiang, Harbin, China; 4 Clinical Research Center for Colorectal Cancer in Heilongjiang, Harbin, China; 5 Radiotherapy Technology Center, Harbin Medical University Cancer Hospital, Harbin, China

**Keywords:** Radiotherapy, Cancer genomics

## Abstract

Radiotherapy is essential in the treatment of colorectal cancer (CRC), but the presence of drug resistance leads to poor prognosis for CRC patients. Identifying targets and mechanisms for regulating radiotherapy resistance has high clinical value. This study identifies CCR4-NOT transcription complex subunit 7 (CNOT7) as a key factor mediating radiotherapy resistance in CRC by stabilizing XRCC6 protein and enhancing non-homologous end joining (NHEJ) mediated DNA damage repair (DDR) pathway. Proteomic analysis of 45 CRC tissues revealed that elevated CNOT7 expression correlates with poorer responses to neoadjuvant radiotherapy and lower disease control rate (DCR). We demonstrated that CNOT7 knockdown enhances radiosensitivity by impairing NHEJ mediated double-strand breaks (DSBs) repair and promoting apoptosis in vitro and in vivo. Mechanistically, CNOT7 interacts with XRCC6 to stabilize its protein levels by inhibiting TRIM21-mediated K48-linked ubiquitination at lysine 526, thereby facilitating efficient DNA repair. CNOT7 accelerates degradation of TRIM21 mRNA through its deadenylase activity. Additionally, the combination of STL127705, an inhibitor of the XRCC6/XRCC5 heterodimer, with radiotherapy notably suppressed tumor growth in patient-derived xenograft (PDX) and cell line mouse transplant tumor models, especially in the context of CNOT7 deficiency. These findings elucidate the function of CNOT7 in promoting DNA repair and radiotherapy resistance in CRC, highlighting that targeting the CNOT7-TRIM21-XRCC6 axis provides a promising therapeutic approach to overcome radiotherapy resistance and improve clinical outcomes for CRC patients.

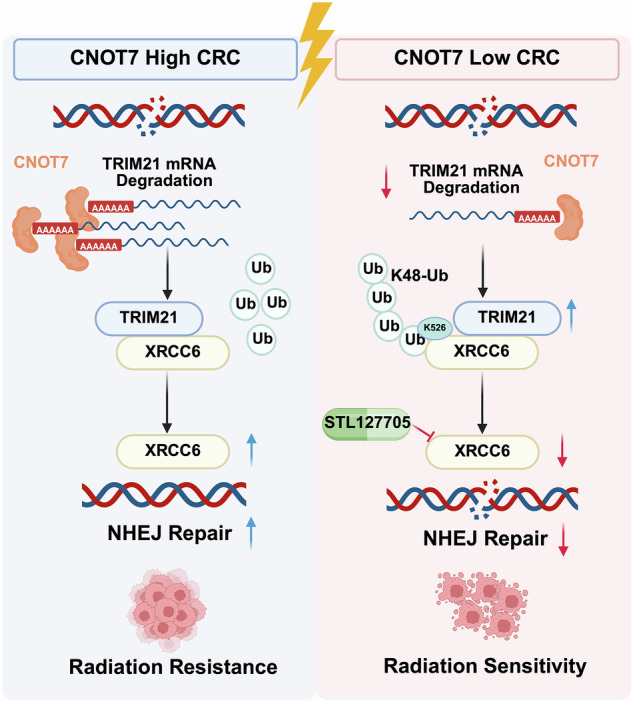

## Introduction

CRC is the third most frequently diagnosed cancer and the second leading cause of cancer-related mortality worldwide [1]. Despite progress in treatment options including immunotherapy and targeted therapies, neoadjuvant radiotherapy remains the standard treatment, particularly for middle to low rectal cancer [2, 3]. However, some patients undergoing radiotherapy fail to achieve effective tumor suppression. This is due to inherent resistance of certain tumor cell subsets to radiotherapy, as well as the development of acquired resistance following repeated radiotherapy sessions. Such resistance significantly worsens the prognosis for rectal cancer patients [4–6]. Unraveling the mechanisms that affect radiotherapy resistance could provide valuable insights and potential strategies for the treatment of CRC.

CNOT7 is a crucial part of CCR4-NOT complex, a multifunctional regulatory complex that is linked to various cellular processes, primarily at post-transcriptional stage. CCR4-NOT complex subunits work synergistically, with specific subunits contributing to distinct functions such as deadenylation, transcriptional regulation, and mRNA decay. As a primary deadenylase subunit, CNOT7 removes the poly(A) tails of mRNA, promoting mRNA decay. This process influences mRNA stability and translation efficiency and regulates gene expression by controlling the half-life of specific transcripts through mRNA degradation [7]. Previous studies have revealed that CNOT7 can regulate ovarian cancer proliferation through the AKT pathway and modulate interferon responses via the JAK-STAT1 pathway [8, 9]. CNOT7 regulates the progression of glioma by affecting the E2F targets, G2/M checkpoint, TNF-α and IL6-JAK-STAT3 signaling pathway through NF-κB, leading to poor prognosis in glioma [10]. In addition, the knockout of CNOT4 can lead to defect in cellular DNA damage repair [11]. However, no studies have explored the relationship between CNOT7 and tumor radiotherapy sensitivity.

Among the factors influencing radiotherapy sensitivity, DNA damage repair mechanisms play a pivotal role [12, 13]. Radiation therapy can induce DNA damage, leading to DSBs, base damage and single strand breaks. Among these, DSBs are most harmful types. The repair of DSBs primarily involves homologous recombination (HR) and NHEJ [14]. HR is a high-fidelity repair process, while NHEJ directly ligates the damaged DNA ends without requiring template and can occur at any phase of the cell cycle. Targeting these pathways is regarded as primary approach to increase tumor sensitivity to radiotherapy. For example, targeting and inhibiting the activity of DNA-PKcs can significantly enhance the sensitivity to radiotherapy [15, 16]. Modulating the ubiquitination of PARP1 can influence the radiotherapy sensitivity of esophageal cancer by affecting the HR repair pathway [17]. Overall, targeting DNA damage repair pathways to regulate tumor radiotherapy sensitivity holds significant research potential in the future.

Ubiquitination primarily governs protein degradation. Researches have revealed that ubiquitination is linked to numerous processes, such as proliferation, division and DNA damage repair [18, 19]. In this process, E3 ubiquitin ligases act as the key rate-limiting enzymes, with the TRIM family being a typical representative of E3 ligases. Tripartite motif containing 21 (TRIM21) is a key protein in the TRIM family [20]. TRIM21 regulates SREBF1 protein stability through ubiquitination to weaken kidney cancer onset [21], and in liver cancer, TRIM21 enhances progression via inhibiting p62-Keap1-Nrf2 pathway [22]. Additionally, TRIM21 can induce c-Myc autophagy and sensitize CRC to regorafenib treatment [23]. Therefore, interfering with the ubiquitination of DNA damage repair proteins to influence radiotherapy sensitivity could be a highly effective therapeutic strategy. However, the connection between CNOT7, DNA damage repair and ubiquitination remains unclear.

Here, we pinpointed CNOT7 as a key protein influencing the radiotherapy sensitivity of rectal cancer through proteomic sequencing of patient tissues. Patients with high CNOT7 expression often exhibit poorer prognoses during radiotherapy. We explored the potential mechanisms by which CNOT7 affects radiotherapy sensitivity in CRC. CNOT7 reduces polyubiquitination of K48 linked lysine 526 on X-ray repair cross complementing 6 (XRCC6) by regulating TRIM21. This enhances DNA damage repair primarily mediated by the NHEJ pathway, thereby conferring radiotherapy resistance to colorectal cancer cells. Our results indicate that targeting CNOT7 could be a promising therapeutic target for CRC.

## Materials and methods

### Patient samples

45 paired CRC tumor tissues before neoadjuvant therapy were acquired from Harbin Medical University Cancer Hospital between 2012 and 2018. Following neoadjuvant therapy, all patients received the standard total mesorectal excision (TME) procedure. Patients were categorized into complete response (CR), partial response (PR), and stable disease (SD) groups, according to extent of tumor regression. CR is identified as complete resolution of target lesions, with short diameter of any pathological lymph nodes reduced to less than 10 mm. PR is characterized by the decrease of at least 30% in total diameter of target lesions compared to the baseline measurement. SD represents a condition where the changes in target lesions do not fulfill the norm for either PR or Progressive Disease (PD). DCR is defined as the percentage of patients whose tumors exhibit CR, PR or SD over a specified duration. Clinical data of CRC patients was provided in Supplementary Table 1. All participants gave written informed consent and the study was approved by the Ethics Committee of Harbin Medical University Cancer Hospital.

### Cell lines

HT29, HCT116, HCT15 and SW480 and 293 T cells were obtained from National Collection of Authenticated Cell Cultures. 293 T were cultured in DMEM and CRC cell lines were cultured in RPMI-1640. The culture medium contained 1% penicillin/streptomycin and 10% fetal bovine serum. Cells were passaged every 2 to 3 days. The identity of all cells was confirmed via short tandem repeat DNA profiling and mycoplasma contamination was excluded using a mycoplasma PCR detection kit.

### Plasmid

Lentiviral pLent-U6-CMV-copGFP-P2A-Puro vector was used to construct shCNOT7 plasmids which were purchased from Weizhen Biosciences Inc (Shandong, China). Sequences were shown below:

shCNOT7-1:5’-GGACTCTATAGAGCTACTAACTTCAAGAGAGTTAGTAGCTCTATAGAGTCCTTTTTT-3’

shCNOT7-2:5’-GCGGTTACGACTTTGGCTACTTTCAAGAGAAGTAGCCAAAGTCGTAACCGCTTTTTT-3

The pCDH-EF1-MCS-CMV-copGFP-T2A-Puro vector was used to construct recombinant overexpression plasmids. Wild-type CNOT7, wild-type TRIM21, His-tagged XRCC6, and XRCC6 mutants (K9R, K238R, K526R) and flag-tagged were cloned into this vector. WT, K63 only, and K48 only plasmids were obtained from Synbio Technologies (Suzhou, China). Small interfering RNA (siRNA) for Trim21 and XRCC6 was obtained from General biol (Anhui, China). Sequences were shown below:

siXRCC6: 5’-AGGAAACAGAAGAGCUAAATT-3’

siTRIM21-1: 5’-GGAAAUUGCAAUAAAGAGATT-3’

siTRIM21-2: 5’-GAGCAUACCUGGAAAUGAATT -3’

siTRIM21-3: 5’-GGAAAUUGCAAUAAAGAGATT-3’.

### Cell transfection

The jetPRIME® transfection reagent (Polyplus, #101000046) was used to cell transfection. 48 hours after transfection, western blotting and Reverse transcription and quantitative Polymerase Chain Reaction (RT-qPCR) were employed to evaluate knockdown and overexpression effect.

### RT-qPCR

Total RNA was isolated from cultured cells and frozen tissues using Trizol reagent. Briefly, 1 mL Trizol was added per sample. Following homogenization, 200 μL chloroform was added, and samples were vortexed vigorously for 30 seconds. After incubation for 10 minutes at room temperature (RT), samples were centrifuged for 10 minutes at 4 °C at 12000 g. The colorless upper aqueous phase was carefully transferred to a new RNase-free tube. RNA was precipitated by adding 400 μL isopropanol, mixing by inversion, and incubating for 10 minutes at RT, followed by centrifugation at 12000 g for 10 minutes at 4 °C. The supernatant was discarded, and the RNA pellet was washed twice with 1 mL 75% ethanol. The pellet was air-dried for 5 minutes and dissolved in DEPC water by repeated pipetting. RNA concentration was determined spectrophotometrically. The RNA was reverse transcribe into CDNA to perform RT-qPCR analysis as previously described (Thermo Fisher, #15596026) [24]. The 2^(-ΔΔCt) method was employed to analyze target gene expression differences in control and experimental groups. Primer sequences were shown below:

H-CNOT7-F: 5′-ATGCCAGCGGCAACTGTAG-3′

H-CNOT7-R: 5′-TCGGTGTCCATAGCAACGTAA-3′

H-XRCC5-F: 5′-GTGCGGTCGGGGAATAAGG-3′

H-XRCC5-R: 5′-GGGGATTCTATACCAGGAATGGA-3′

H-XRCC6-F: 5′-GTTGATGCCTCCAAGGCTATG-3′

H-XRCC6-R: 5′-CCCCTTAAACTGGTCAAGCTCTA-3′

H-PRKDC-F: 5′-CCTGGCCGGTCATCAACTG-3′

H-PRKDC-R: 5′-AGTAAGGTGCGATCTTCTGGC-3′

H-MRE11-F: 5′-ATGCAGTCAGAGGAAATGATACG-3′

H-MRE11-R: 5′-CAGGCCGATCACCCATACAAT-3′

H-RAD50-F: 5′-TTTGGTTGGACCCAATGGGG-3′

H-RAD50-R: 5′-CAGGAGGGAAATCTCCAGTACAA-3′

H-TRIM21-F: 5′-TCAGCAGCACGCTTGACAAT-3′

H-TRIM21-R: 5′-GGCCACACTCGATGCTCAC-3′

H-ACTIN-F: 5′-CATGTACGTTGCTATCCAGGC-3′

H-ACTIN-R: 5′-CTCCTTAATGTCACGCACGAT-3′.

### Western blotting

Tissues and cells were lysed on ice for 30 minutes in RIPA buffer (Thermo Fisher, #89001). Lysates were centrifuged for 10 minutes at 12000 rpm and supernatant was mixed with SDS loading buffer (Beyotime, #P0015F). Proteins were separated by SDS-PAGE, transferred to PVDF, blocked and finally incubated overnight with antibodies at 4 °C. The antibodies were: CNOT7 (Proteintech, #14102-1-AP, RRID:AB_2245087), XRCC5 (Proteintech, #16389-1-AP, RRID:AB_2257509), Phospho-DNA-Pkcs (Ser2056) (CST, #68716, RRID:AB_2939025), XRCC6 (Proteintech, #10723-1-AP, RRID:AB_2218756), DNA-Pkcs (Proteintech, #19983-1-AP, RRID:AB_10642811), RAD50(Proteintech, #29390-1-AP, RRID:AB_2918289), NBS1(Proteintech, # 55025-1-AP, RRID:AB_10858928), Phospho-Histone H2A.X (Ser139) (20E3) (CST, #9718, RRID:AB_2118009), TRIM21 (Proteintech, #12108-1-AP, RRID:AB_2209469), RAD51(Proteintech, #14961-1-AP), 53BP1(Proteintech, # 20002-1-AP, RRID:AB_3085592), MRE11(Proteintech,#10744-1-AP,RRID:AB_2145118) and Ub (Proteintech, #10201-2-AP, RRID:AB_671515).

### Cell proliferation

Cells were seeded at 1000 cells/ well onto 96-well plates. If radiation therapy is needed, 2, 4, 6 or 8 Gy X-ray will be administered. CCK8 (GLPBIO, #GK10001) was performed following manufacturer’s guidelines.

### Colony formation assay

Cells were seeded in 6-well plates. 8 Gy X-ray was administered in radiation treatment group, followed by incubation for 21 days. Subsequently, cells were fixed with methanol and stained with crystal violet (Solarbio, #C8470) for colony counting.

### Immunohistochemistry (IHC) staining

CRC tissue and mouse transplanted tumors were dehydrated and embedded and cut into 4 mm sections. Sections undergo deparaffinization, antigen repair, and sealing treatment. Next, the sections were incubated with CNOT7, TRIM21, XRCC6 and 53BP1 antibodies overnight at 4 °C. IHC score was calculated based on the intensity of staining (0, negative staining; 1, weak staining; 2, moderate staining; 3, strong staining) and the percentage of positively stained cells (0, 0–5%; 1, 5–25%; 2, 26–50%; 3, 51–75%; 4, 76–100%). A final score of ≥6 was classified as high expression, while scores <6 were considered low expression.

### HR, NHEJ reporter assays

The EJ5-GFP and DR-GFP reporter plasmids were obtained from Vigene Bio (Shandong, China). Cells were transfected with the EJ5-GFP reporter plasmids and DR-GFP followed by incubation for 24 hours. Then, I-SceI plasmid was added in cells. 48 hours later, the proportion of GFP+ cells was accessed by flow cytometry. The DNA repair efficiency mediated via NHEJ and HR was evaluated by Flow Jo software.

### Immunofluorescence

Cells were fixed with 4% paraformaldehyde at RT for 15 minutes and permeabilized with 1% Triton X-100 in PBS for 5 minutes. After three additional PBS washes, cells were blocked with 5% BSA at RT for 1 hour and primary antibodies were incubated overnight at 4 °C. Afterward, the cells were incubated with fluorescent secondary antibody and stained with DAPI (Beyotime, #P0131), followed by sealing to prevent fluorescence quenching. Finally, fluorescent images were captured using Leica confocal fluorescence microscope.

### Neutral comet assay

Neutral comet assay was conducted using Beyotime Comet Assay Kit. A mixture of 10 μL of the cell suspension (approximately 1000 cells) and 0.7% low-melting-point agarose from the kit was transferred onto comet assay slides. The slides were immersed in pre-chilled neutral lysis buffer with DMSO and incubated for 1–2 hours at 4 °C. The slides then were electrophoresed under neutral conditions at 25 V for 20–30 minutes. Slides were then immersed in neutral buffer for neutralization three times at 4 °C, with each neutralization lasting 5 minutes. Finally, propidium iodide solution was added to slides for staining for 15 minutes. The images were captured using fluorescence microscope, and comet tail moment was accessed using ImageJ.

### Cell apoptosis

Cell apoptosis was detected using Annexin V-FITC Apoptosis Detection Kit (Beyotime, #C1062S). Cells treated with or without radiotherapy were mixed with PI and Annexin V, incubated at room temperature for 20 minutes, detected using flow cytometry, and analyzed by Flow Jo software.

### Immunoprecipitation-mass spectrometry (IP-MS)

Cells were lysed using IP lysis buffer (Thermo Scientific™, #87787) on ice for 30 minutes and lysates were collected by centrifugation. The lysates were incubated with antibodies at 4 °C. Beads (Thermo Scientific™, #88802) were added to the lysates. The immunoprecipitates were harvested and resuspended in IP lysis buffer, SDS loading buffer (Beyotime, #P0015F) was added to the mixture and the mixture was boiled at 100 °C for 10 minutes. Then WB was performed and the WB gel strip samples were sent to PTM Biolab (Hangzhou, China) for mass spectrometry analysis. The gel pieces were enzymatically digested into peptides. The peptides were dissolved in the liquid chromatography mobile phase A and then separated using the EASY-nLC 1000 ultra-high performance liquid chromatography system. After separation, it was injected into an NSI ion source for ionization and analyzed using a Q ExactiveTM Plus mass spectrometer. The resulting MS/MS data were processed using Proteome Discoverer 1.3. The raw data of IP-MS can be found in Supplementary Table 2.

### RNA immunoprecipitation assays

1 × 10⁷ cells were fixed with 1% formaldehyde for 15 min, followed by quenching with 1 M glycine for 5 min to terminate fixation. After PBS washes, cells were scraped and lysed in lysis buffer (20 mM Tris-Cl [pH 8.0], 0.5% NP-40, 1 mM EDTA, 10 mM NaCl) supplemented with RNasin and protease. Lysates were subjected to IP using anti-CNOT7 antibody or IgG antibody, pre-coupled to Dynabeads Protein A. The immunoprecipitates were washed. qRT-PCR was used to detect and analyze bound RNA.

### Ubiquitination assay

Cells were lysed using RIPA lysis buffer. Following the IP method described earlier, cell lysates were subjected to immunoprecipitated, followed by western blotting to access ubiquitination levels.

### Mouse xenograft model

NYG and nude mice were purchased from Changsheng Biotechnology (Liaoning, China). We obtained approval from Institutional Animal Care and Use Committee of Harbin Medical University and followed ARRIVE guidelines.

A suspension of 1 × 10^6^ HCT116 cells was injected into the right flank of mice to establish transplanted tumor model. Approximately 7 days post-injection, tumor volume was accessed using calipers. Tumor size was calculated by formula: V = a2×b/2, where a represents the short axis and b represents the long axis of the tumor. On day 13 post-tumor formation, mice received radiotherapy (6 Gy per day for 3 days). STL127705 was intraperitoneally injected at a dose of 50 mg/kg. Finally, mice were euthanized, and tumor volume and weight were accessed.

PDX models were established using CRC tissues obtained from patients who underwent surgical resection. Tumor tissues were cut into small ieces and implanted into 6-week-old female NYG mice. Tumor regions were subjected to 6 Gy of fractionated X-ray irradiation or combined treatment with STL127705, administered via intraperitoneal injection. Tumor progression was accessed through by tumor volume and tumor weight.

### 4D-FastDIA quantitative proteomics

This sequencing service was provided by Hangzhou Jingjie Biotechnology Co., Ltd. After deparaffinization, the protein samples were transferred into 1.5 mL centrifuge tubes and mixed with lysis buffer (1% SDC, 1% protease inhibitor), followed by ultrasonic disruption. Equal amounts of protein were then taken for digestion. Dithiothreitol (DTT) was added to a final concentration of 5 mM, and the samples were reduced at 56 °C for 30 minutes. Iodoacetamide was then added, and the samples were incubated in the dark at room temperature for 15 minutes. Finally, trypsin was added for enzymatic digestion. The resulting products were analyzed by liquid chromatography-tandem mass spectrometry (LC-MS/MS).

### Statistical analysis

Quantitative data are showed as mean ± SEM. Differences between samples were evaluated using Fisher’s exact test, Student’s t-test and Fisher’s analysis of variance. Log-rank test was used to survival analysis. All statistical analyses were performed using GraphPad Prism software. Statistical significance was determined as follows: *p < 0.05, **p < 0.01, ***p < 0.001, and ****p < 0.0001.

## Results

### CNOT7 is linked to radiation resistance and indicates a poor prognosis in CRC

**Fig. 1:**
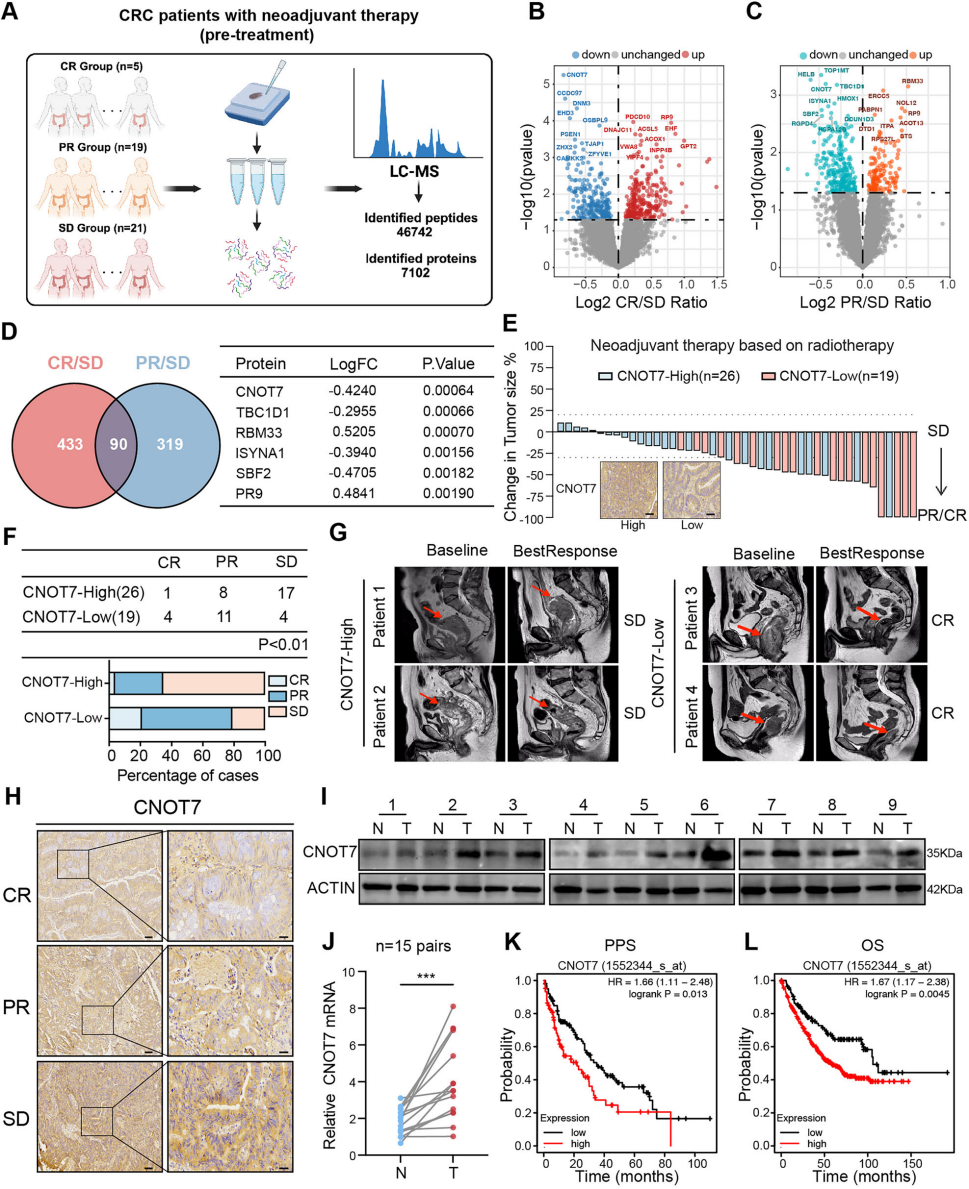
CNOT7 is linked to radiation resistance and indicates a poor prognosis in CRC. **A** Schematic diagram of pre-treatment tissue proteome sequencing for CRC patients receiving neoadjuvant therapy. **B** Volcano diagram of differential proteins between CR group and SD group patients. **C** Volcano diagram of differential proteins between PR group and SD group patients. **D** The Venn diagram shows the common differential proteins between the CR/SD group and the PR/SD group. **E** Waterfall plot shows the changes in tumor burden of CRC patients undergoing radiotherapy from initiation to the best objective response. Patients were grouped based on the expression of CNOT7 in tumor tissue. Scale bar, 120 μm. **F** Table and histogram recapitulating the outcomes of 45 CRC patients who received radiotherapy. **G** Representative magnetic resonance (MR) images of the rectum showing tumor changes at baseline and the best response after radiotherapy. Red arrows indicate the lesions. The panels display the CNOT7 status and therapeutic evaluation. **H** Representative immunohistochemical images of CNOT7. Right scale bar, 100 μm. Left scale bar, 30 μm. **I** Western blotting of CNOT7 in CRC and adjacent normal tissues. **J** CNOT7 mRNA levels in CRC and adjacent normal tissues. **K**, **L** Kaplan-Meier curves were generated on the www.kmplot.com website to show the post-progression survival (**K**) and overall survival (**L**) in III and IV stage CRC patients with low or high CNOT7 expression.

To identify critical genes that affect sensitivity of CRC to radiotherapy, we collected 45 rectal cancer tissues before neoadjuvant therapy. The patients were grouped according to the degree of tumor regression after neoadjuvant therapy, including 5 CR, 19 PR, and 21 SD patients. Subsequently, proteomic analysis was performed on these tissues and 7102 proteins were identified (Fig. 1A). We analyzed the differential proteins between CR and SD group, and between PR and SD group, and generated volcano plots (Fig. 1B, C). We identified the overlapping differentially expressed proteins between two groups and screened out 90 proteins, with CNOT7 showing the most significant difference (Fig. 1D). Approximately 42.2% (19 out of 45) of the enrolled patients had lower CNOT7 protein abundance in their tumors and most patients in the CNOT7 low-expression group achieved CR or PR after neoadjuvant therapy (Fig. 1E). In the CNOT7 low-expression group, DCR was 78.9% (15 out of 19), which was higher than DCR of CNOT7 high-expression group (DCR, 34.6%, 9 out of 26; P < 0.01) (Fig. 1F). Consistently, representative cases of rectal magnetic resonance imaging (MRI) showed tumor shrinkage after radiotherapy in patients with CNOT7 low expression, whereas tumors in patients with CNOT7 high expression exhibited stable disease (Fig. 1G). We further accessed CNOT7 levels in rectal cancer tissues before radiotherapy and compared the treatment outcomes. The CNOT7 expression was higher in patients who were insensitive to radiotherapy (Fig. 1H and Fig. S1A). In summary, these results revelated that high CNOT7 expression in tumor tissues was linked to resistance to radiotherapy. We revealed that CNOT7 protein and mRNA expression were higher in CRC tissues (Fig. 1I, J). Immunohistochemistry also indicated that CNOT7 was higher in tumor tissues (Fig. S1B). In other tumors, mRNA level of CNOT7 is also higher in tumor tissues (Fig. S1C–H). Compared to NCM460 intestinal epithelial cells, the mRNA and protein level of CNOT7 is also higher in CRC cell lines (Fig. S1I, J). Kaplan-Meier curves of survival revealed that CNOT7 high-expression CRC patients in III and IV stage tend to have worse PPS and OS (Fig. 1K, L).

### CNOT7 knockdown promotes sensitivity of CRC to radiotherapy in vitro and in vivo

**Fig. 2:**
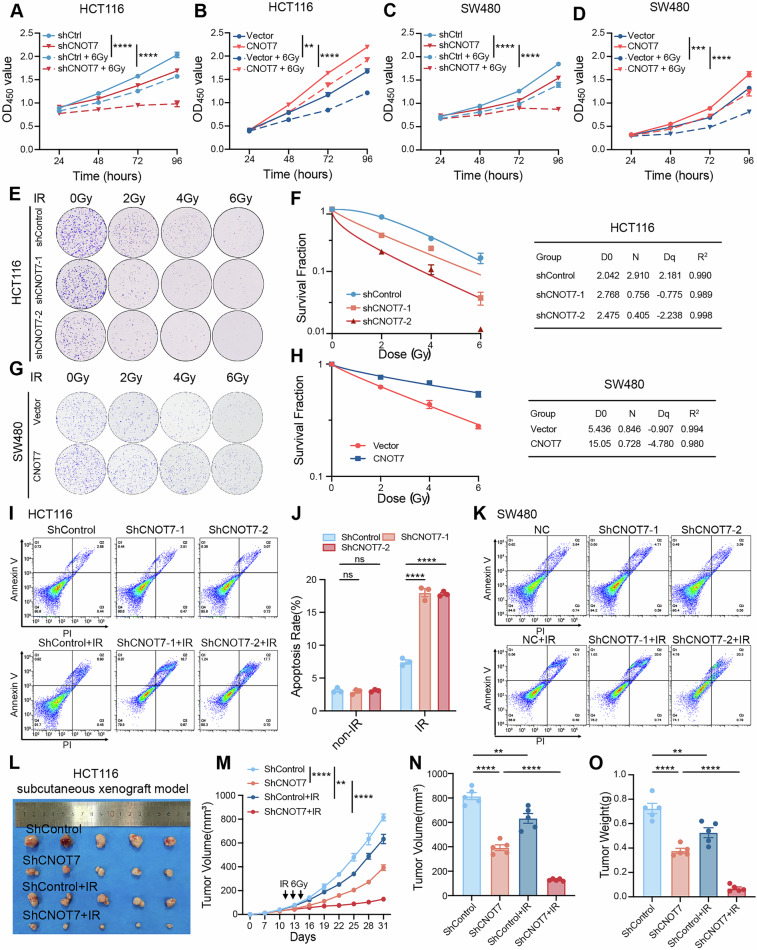
CNOT7 knockdown promotes sensitivity of CRC to radiotherapy in vitro and in vivo. **A**–**D** CCK8 assay revealed that CNOT7 knockdown increased the radiation therapy sensitivity of CRC cells and CNOT7 overexpression promoted CRC cell resistance to radiation therapy. **E**, **F** Representative images and corresponding survival fraction curves of colony formation assays showed that CNOT7 knockdown increased radiotherapy sensitivity in HCT116. **G**, **H** Representative images and corresponding survival fraction curves of colony formation assays showed that overexpression of CNOT7 promotes radiation resistance in SW480. **I**, **J** Flow cytometry was employed to access the proportion of apoptosis in HCT116 and CNOT7 knockdown HCT116 cells with or without radiotherapy. **K** Flow cytometry was used to evaluate the proportion of apoptosis in SW480 and CNOT7 knockdown SW480 cells with or without radiotherapy. **L** Gross images of HCT116 and CNOT7 knockdown HCT116 cell-derived subcutaneous tumors with or without radiotherapy. **M**–**O** Tumor growth curves (**M**), tumor volume (**N**) and tumor weights (**O**) of HCT116 and CNOT7 knockdown HCT116 cell-derived subcutaneous tumors with or without radiotherapy.

We detected the levels of CNOT7 in various colorectal cancer cell lines using western blotting and RT-qPCR, and selected HCT116 and SW480 cells for further experiments (Fig. S1I, J). HCT116 cells are colon cancer cells, and SW480 cells are rectal cancer cells. To investigate whether CNOT7 affects radiosensitivity, we constructed stable HCT116 and SW480 cell lines with CNOT7 overexpression and knockdown. Western blotting and RT-qPCR were performed to detect CNOT7 level (Fig. S2A–H). CNOT7 knockdown in HCT116 showed slower wound healing, less migration and invasion compared to control (Fig. S2I–M). We evaluated the effect of CNOT7 on radiosensitivity. The outcomes revelated that CNOT7 knockdown inhibited proliferation and colony formation of CRC cells after radiotherapy, while CNOT7 overexpression significantly promoted colony formation and cell survival following radiation exposure (Fig. 2A–H and Fig. S3A–D). By flow cytometry, we observed that the apoptosis rate was significantly increased in CNOT7 knockdown cells after radiation exposure in HCT116 and SW480 (Fig. 2I–K and Fig. S3E). To investigate whether CNOT7 knockdown enhances the CRC radiosensitivity in vivo, we constructed a mouse transplant tumor model. We discovered that CNOT7 knockdown tumors had slower tumor growth rate, smaller volume and weight and was sensitive to radiation treatment compared to the HCT116 tumors (Fig. 2L–O). Overall, these results indicate that tumors in the CNOT7 knockdown group showed enhanced sensitivity to radiotherapy.

### CNOT7 knockdown impairs NHEJ mediated DSB repair

**Fig. 3:**
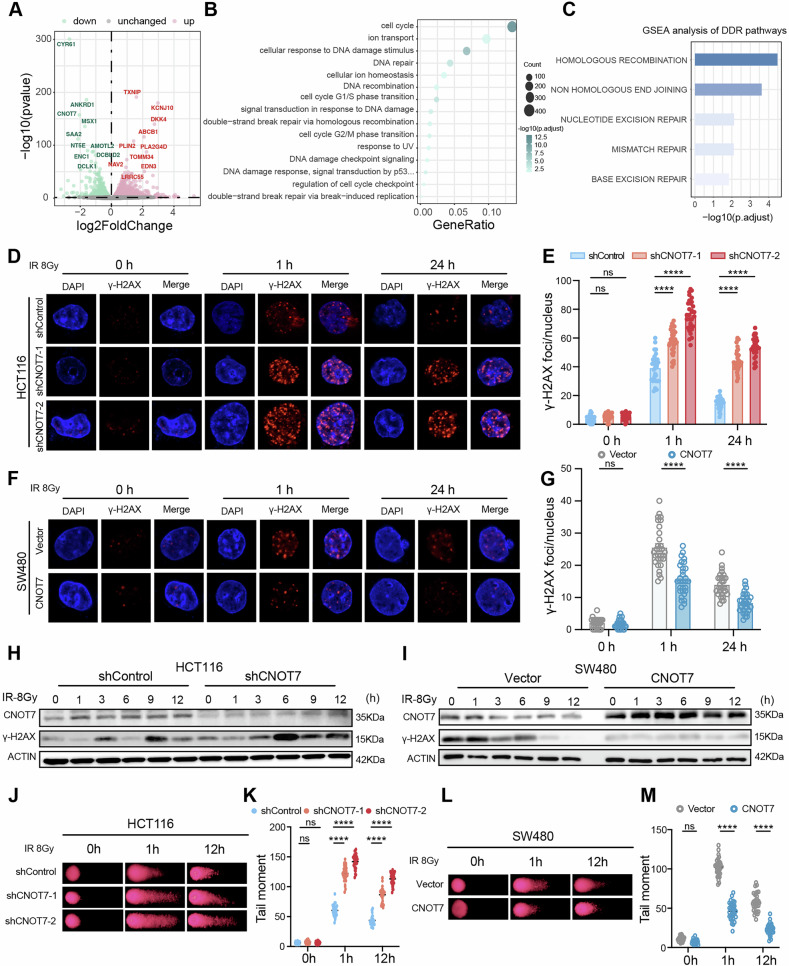
CNOT7 knockdown impairs the DNA damage repair function. **A** The volcano plot shows the differential gene expression between HCT116 and CNOT7 knockdown HCT116 cells in RNA-seq analysis. **B** GO analysis was performed on differential genes. **C** GSEA analysis of DNA damage repair-related pathways. **D**, **E** Representative images and quantitation of γH2AX positive nuclei in HCT116 and CNOT7 knockdown HCT116 cells at different time points. **F**, **G** Representative images and quantitation of γ-H2AX positive nuclei in SW480 and CNOT7 overexpression SW480 cells at different time points. **H** Western blotting of CNOT7 and γ-H2AX protein expression at different time points after radiation treatment in HCT116 and CNOT7 knockdown HCT116 cell lines. **I** Western blotting of CNOT7 and γ-H2AX protein expression at different time points after radiation treatment in SW480 and CNOT7 overexpression SW480 cell lines. **J**, **K** Representative images of comet assay and quantitative analysis of tail moment in HCT116 and CNOT7 knockdown HCT116 cells at different time points. **L**, **M** Representative images of comet assay and quantitative analysis of tail moment in SW480 and CNOT7 overexpression SW480 cells at different time points.

To investigate the role of CNOT7 in regulating radiotherapy sensitivity, RNA sequencing was conducted on HCT116 and HCT116 with CNOT7 knockdown, identifying 1530 increased genes and 1161 decreased genes (Fig. 3A). These differentially expressed genes were further analyzed by GO and GSEA. GO analysis revealed that the enrichment of double-strand break repair related pathways, such as cellular response to DNA damage stimulus, DNA repair, double-strand break repair via homologous recombination, DNA damage response, double-strand break repair via break-induced replication (Fig. 3B). To characterize the roles of CNOT7 in regulating DDR, we implemented GSEA and found that HR and NHEJ pathways involved in DNA double-strand break repair were significantly regulated (Fig. 3C). γ-H2AX is recognized as a biomarker for DSBs, the most fatal lesions caused by radiotherapy and is linked to HR and NHEJ repair. Therefore, we investigated the CNOT7 effect on radiotherapy-induced γ-H2AX focus formation. The results indicated that the accumulation of γ-H2AX increased after 1 hour and 24 hours of radiotherapy in CNOT7 knockdown CRC cells (Fig. 3D, E and Fig. S4A, B), while the accumulation of γ-H2AX was notably reduced in CNOT7 overexpressing cells (Fig. 3F, G and Fig. S4C, D). Then we treated CNOT7 knockdown and overexpression CRC cell lines with radiotherapy and then measured the levels of CNOT7 and γ-H2AX proteins at different time points to monitor cell DNA damage. We found that 1 to 12 hours after radiation exposure, the γ-H2AX levels were higher in the CNOT7 knockdown CRC cells, while the γ-H2AX levels were lower in the CNOT7 overexpression CRC cells (Fig. 3H, I). Comet assay showed that CNOT7 knockdown significantly increased tail distance after radiotherapy in CRC cells (Fig. 3J, K and Fig. S4E, F), indicating a higher degree of DNA double-strand damage in CNOT7 knockdown cells. However, CNOT7 overexpression enhanced DDR capacity and shortened the tail distance after radiotherapy in CRC cells (Fig. 3L, M and Fig. S4G, H).

**Fig. 4:**
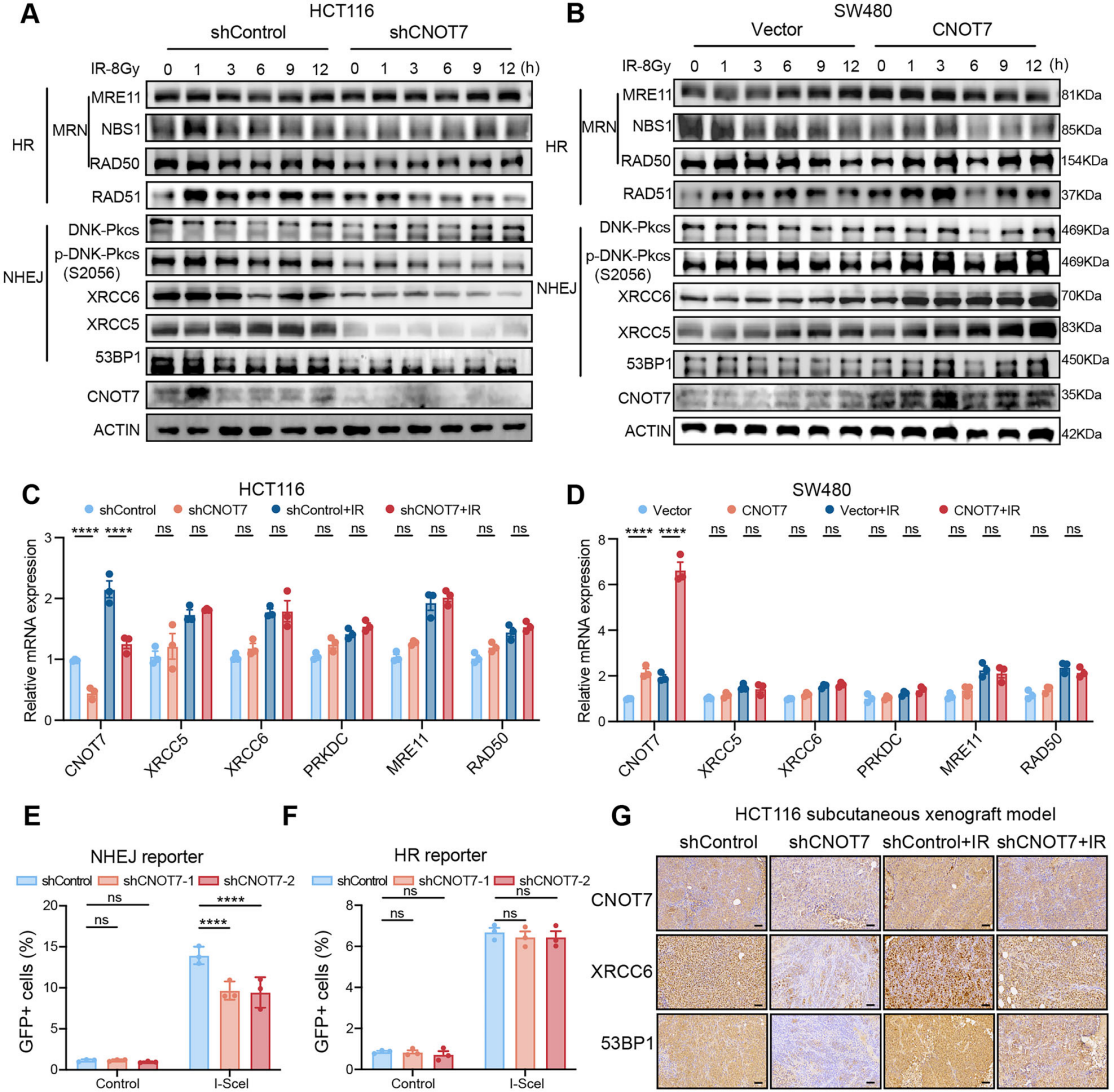
CNOT7 knockdown impairs NHEJ mediated DSB repair. **A** Western blotting of HR and NHEJ related protein expression at different time points after radiation treatment in HCT116 and CNOT7 knockdown HCT116 cell lines. **B** Western blotting of homologous recombination (HR) and non-homologous end joining (NHEJ) related protein expression at different time points after radiation treatment in SW480 and CNOT7 overexpression SW480 cell lines. **C** RT-qPCR analysis of the expression levels of homologous recombination (HR) and non-homologous end joining (NHEJ) related genes in HCT116 and CNOT7 knockdown HCT116 cell lines. **D** RT-qPCR analysis of the expression levels of homologous recombination (HR) and non-homologous end joining (NHEJ) related genes in SW480 and CNOT7 overexpression SW480 cell lines. **E**, **F** NHEJ and HR repair efficiency were assessed in HCT116 cells transfected with DR-GFP and EJ5-GFP reporter plasmids. **G** Representative immunohistochemistry (IHC) images showing the expression of CNOT7, XRCC6, and 53BP1 in HCT116 and CNOT7 knockdown HCT116 cell-derived subcutaneous tumors with or without radiotherapy. Scale bar: 60 μm.

HR and NHEJ are the two primary DNA repair mechanisms [25, 26]. To determine which pathway CNOT7 primarily affects in CRC cells, we examined the proteins levels related to NHEJ and HR in CRC cells. We found that CNOT7 knockdown cells exhibited low expression of NHEJ-related proteins, including p-DNA-Pkcs (S2056), XRCC5, XRCC6, and 53BP1, while these proteins were highly expressed in the CNOT7 overexpression group. In contrast, changes in HR-related proteins were not significant (Fig. 4A, B). To investigate whether CNOT7 affects the transcription of NHEJ-related genes, we measured relevant mRNA levels in CNOT7 knockdown and overexpression cells after radiotherapy. We found that mRNAs levels did not show significant differences (Fig. 4C, D). Subsequently, we used EJ5-GFP and DR-GFP reporter gene assays to assess efficiency of NHEJ and HR. Flow cytometry analysis showed that NHEJ repair pathway was notably inhibited in CNOT7 knockdown cells, but not the HR pathway (Fig. 4E, F). Immunohistochemical revealed a notably decrease in protein levels of XRCC6 and 53BP1 in CNOT7 knockdown transplanted tumor (Fig. 4G). These findings indicate that CNOT7 enhances DDR through the NHEJ pathway, thereby inducing radiation resistance in CRC cells.

### CNOT7 knockdown promotes degradation of XRCC6 by enhancing ubiquitination

**Fig. 5:**
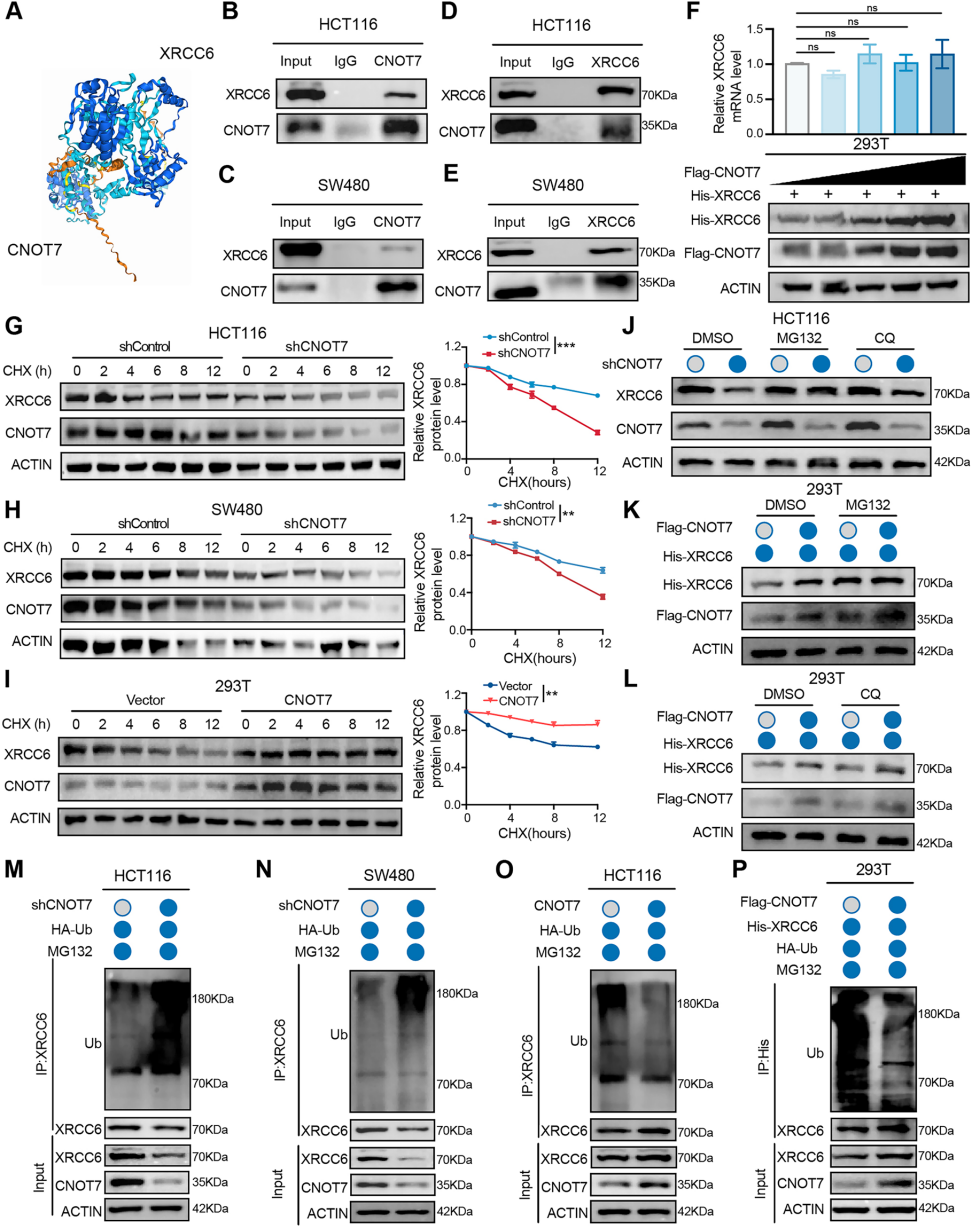
CNOT7 knockdown promotes degradation of XRCC6 by enhancing ubiquitination. **A** Prediction of the interaction between XRCC6 and CNOT7 using AlphaFold 3. **B**, **C** Co-immunoprecipitation (Co-IP) analysis of CNOT7-XRCC6 interaction in HCT116 and SW480 cells. Lysates immunoprecipitated with IgG and anti-CNOT7 antibody. Immunoblots probed with Anti-XRCC6 and Anti-CNOT7 antibodies. **D**, **E** Co-IP analysis of CNOT7-XRCC6 interaction in HCT116 and SW480 cells. Lysates immunoprecipitated with IgG and anti-XRCC6 antibody. Immunoblots probed with Anti-XRCC6 and Anti-CNOT7 antibodies. **F** Detection of XRCC6 protein and RNA levels under CNOT7 overexpression in 293 T cells. **G** Western blotting of XRCC6 expression in HCT116 and CNOT7 knockdown HCT116 cells treated with cycloheximide (CHX 100 µg/ml). **H** Western blotting of XRCC6 expression in SW480 and CNOT7 knockdown SW480 cells treated with cycloheximide (CHX 100 µg/ml). **I** Western blotting of XRCC6 in 293 T and CNOT7 overexpression 293 T cells treated with cycloheximide (CHX 100 µg/ml). **J** Western blotting of XRCC6 and CNOT7 expression in HCT116 and CNOT7 knockdown HCT116 cells treated with MG132 (10 µM) or CQ (50 µM). **K** Western blotting of XRCC6 and CNOT7 in 293 T cells treated with MG132 (10 µM). **L** Western blotting of XRCC6 and CNOT7 expression in 293 T cells treated with CQ (50 µM). **M** Ubiquitination assay of XRCC6 performed in HCT116 and CNOT7 knockdown HCT116 cells. **N** Ubiquitination assay of XRCC6 performed in SW480 and CNOT7 knockdown SW480 cells. **O** Ubiquitination assay of XRCC6 performed in HCT116 and CNOT7 overexpression HCT116 cells. **P** Ubiquitination assay of XRCC6 performed in 293 T cells.

The previous results indicated that CNOT7 knockdown could down-regulate the XRCC6-mediated NHEJ pathway. To further verify the relationship between CNOT7 and XRCC6, we used AlphaFold3 for prediction, and the results showed that CNOT7 and XRCC6 exhibited a high degree of interaction (Fig. 5A). Subsequently, we performed Immunoprecipitation using an anti-CNOT7 antibody to capture XRCC6, and then performed pull-down analysis using anti-XRCC6 antibody to capture CNOT7. The interaction between CNOT7 and XRCC6 was confirmed in both HCT116 and SW480 cells (Fig. 5B–E). We next investigated whether CNOT7 influences XRCC6 expression. Our results indicated that CNOT7 overexpression enhanced the XRCC6 protein levels in a dose-dependent manner, while having no impact on XRCC6 mRNA levels in 293 T (Fig. 5F). In addition, we employed CHX to investigate the impact of CNOT7 on degradation of XRCC6 protein. CHX, an inhibitor of protein synthesis, was used to block new protein synthesis so that the degradation of existing proteins can be studied. Results indicated that after CHX treatment, CNOT7 knockdown significantly increased the degradation of endogenous XRCC6 protein (Fig. 5G, H), while CNOT7 overexpression notably inhibited the degradation of exogenous XRCC6 protein (Fig. 5I). These findings suggest that CNOT7 extends the half-life of XRCC6 protein. We hypothesize that CNOT7 may regulate XRCC6 at post-transcriptional level.

To clarify whether CNOT7 regulates XRCC6 degradation via proteasomal or lysosomal pathway, we treated CNOT7 knockdown HCT116 with MG132 or CQ. MG132, an inhibitor of the ubiquitin-proteasome pathway, was used to determine if a protein is degraded via the proteasomal pathway. CQ, a lysosomal inhibitor that prevents autophagic degradation, was used to determine if a protein is degraded via lysosomal pathway. The results indicated that treatment with MG132 prevented the XRCC6 degradation caused by CNOT7 knockdown (Fig. 5J). Additionally, CQ treatment had no impact on XRCC6 protein levels by CNOT7 in 293 T cells, while MG132 treatment significantly enhanced the inhibition of CNOT7 on XRCC6 degradation (Fig. 5K, L). These findings indicate that CNOT7 limits XRCC6 degradation via the ubiquitin-proteasome pathway. Subsequently, we examined the impact of CNOT7 on XRCC6 ubiquitination. CNOT7 knockdown significantly promoted the ubiquitination of XRCC6 in CRC cell lines (Fig. 5M, N), while CNOT7 overexpression markedly reduced XRCC6 ubiquitination levels (Fig. 5O). Similarly, exogenous expression of CNOT7 also significantly decreased the ubiquitination of exogenous XRCC6 in 293 T (Fig. 5P). These findings indicate that CNOT7 inhibits the ubiquitination of XRCC6 via ubiquitin-proteasome pathway, preventing its protein degradation.

### CNOT7 regulates TRIM21 to deubiquitylate and stabilize XRCC6

**Fig. 6:**
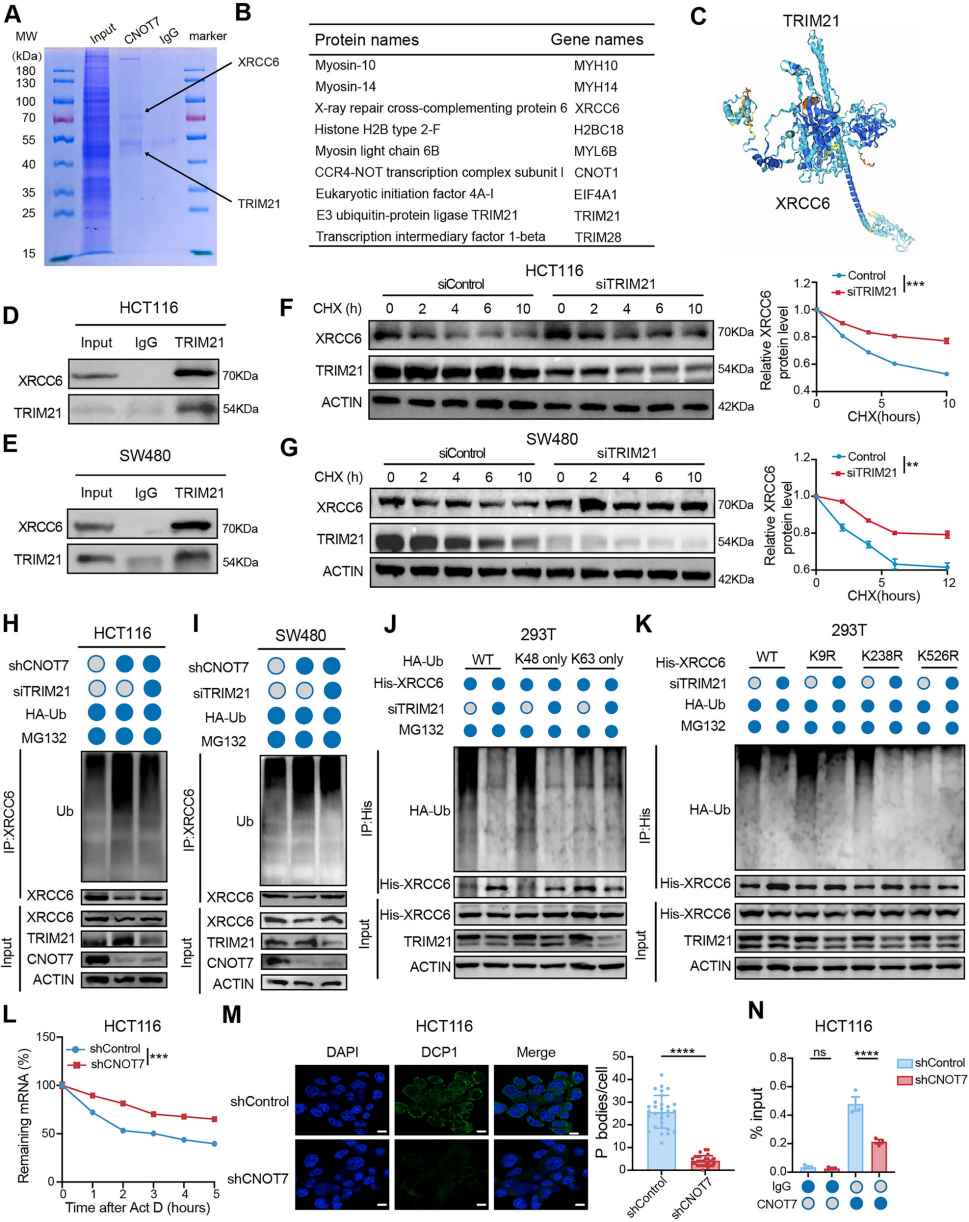
CNOT7 regulates TRIM21 to deubiquitylate and stabilize XRCC6. **A** Immunoprecipitation-Mass Spectrometry (IP-MS) of IgG and CNOT7 antibody in HCT116. **B** The interacting proteins identified by IP-MS analysis. **C** Prediction of the interaction between XRCC6 and TRIM21 using AlphaFold 3. **D**, **E** Co-IP analysis of TRIM21-XRCC6 interaction in HCT116 and SW480 cells. Lysates immunoprecipitated with IgG and anti-TRIM21 antibody. Immunoblots probed with Anti-XRCC6 and Anti-TRIM21 antibodies. **F** Western blotting of XRCC6 expression in HCT116 and TRIM21 knockdown HCT116 cells treated with cycloheximide (CHX 100 µg/ml). **G** Western blotting of XRCC6 expression in SW480 and TRIM21 knockdown SW480 cells treated with cycloheximide (CHX 100 µg/ml). **H** Ubiquitination assay of XRCC6 performed in HCT116, CNOT7 knockdown HCT116 and CNOT7 and TRIM21 knockdown HCT116 with TRIM21 knockdown cells. **I** Ubiquitination assay of XRCC6 performed in SW480, CNOT7 knockdown SW480 and CNOT7 and TRIM21 knockdown SW480 with TRIM21 knockdown cells. **J** Ubiquitination assay of XRCC6 performed in 293 T cells co-transfected with HA-Ub, HA-Ub (K48 only), His-Ub (K63 only), His-XRCC6, siTRIM21, or empty vector plasmids. **K** Ubiquitination assay of XRCC6 performed in 293 T cells co-transfected with His-XRCC6, His-XRCC6 (K9R), His-XRCC6 (K238R), His-XRCC6 (K526R), HA-Ub, siTRIM21, or empty vector plasmids. **L** HCT116 and CNOT7 knockdown HCT116 cells were treated with Actinomycin D (5ug/ml) and mRNA was isolated at the indicated time points. Th RNA decay rate was calculated based on the remaining mRNA levels at each time point relative to time zero. **M** Analysis of P-body formation upon CNOT7 knockdown using an anti-Dcp1a rabbit antibody. Scale bar, 10 μm. **N** RIP-qPCR analysis of the association between CNOT7 and TRIM21 mRNA.

To explore the mechanism of CNOT7 regulating XRCC6, we employed IP-MS to identify potential interaction partners of CNOT7 (Fig. 6A). We found XRCC6 was present among the identified proteins, which verified the previous interaction results between CNOT7 and XRCC6 (Fig. 6B). Based on the mass spectrometry interaction results, we only identified two members of the TRIM family related to ubiquitination, TRIM21 and TRIM28 (Fig. 6B). Because the number of peptides and peptide spectrum matches (PSMs) related to TRIM21 are higher than those of TRIM28, TRIM21 was chosen for research (TRIM21: 5 peptides and 6 PSMs; TRIM28: 1 peptide and 1 PSMs). Therefore, we hypothesize that TRIM21 is linked to ubiquitination and degradation of XRCC6 mediated by CNOT7. We used AlphaFold 3 to predict the interaction ability between TRIM21 and XRCC6 (Fig. 6C). TRIM21 was identified to bind with XRCC6 in CRC cell lines (Fig. 6D, E). Meanwhile, CNOT7 knockdown downregulates XRCC6 expression and upregulates TRIM21 expression in SW480 cell line (Fig. S5A). Moreover, XRCC6 expression increases with TRIM21 knockdown (Fig. S5B). We further examined whether TRIM21 influences protein stability of XRCC6. Our results showed that, after CHX treatment, TRIM21 knockdown notably extended the half-life of XRCC6 protein, suggesting that TRIM21 promotes XRCC6 protein degradation (Fig. 6F, G). Ubiquitination results revealed that CNOT7 knockdown notably enhanced ubiquitination level of endogenous XRCC6, while TRIM21 knockdown could reverse this process (Fig. 6H, I). K63 and K48-linked ubiquitin chains are primary forms of polyubiquitination [27]. K63-linked ubiquitin chains plays a non-degradative role in processes like DDR or NF-κB signaling, whereas K48-linked ubiquitin chains primary marks proteins for degradation through proteasomal pathway [28, 29]. To identify the ubiquitination forms of TRIM21 mediated XRCC6, we transfected WT, K48-only and K63-only vectors into 293 T cells. We found that TRIM21 knockdown reduced XRCC6 degradation mediated by K48-linked polyubiquitination, rather than K63-linked polyubiquitination (Fig. 6J). Next, we predicted the ubiquitination modification site of XRCC6, constructed three XRCC6 lysine/arginine (K/R) substitution mutants (K9R, K238R, and K526R) and transfected them into 293 T. Ubiquitination assays showed that TRIM21 knockdown eliminated the ubiquitination activity of XRCC6-WT, K9R, and K238R mutants, but had no effect on ubiquitination of XRCC6-K526R mutant (Fig. 6K). The elevated TRIM21 protein promotes XRCC6 degradation via K48-linked polyubiquitination at Lys526 site. To investigate whether the XRCC6 K526R mutant affects XRCC6 stability, we performed western blotting assays in HCT116 and SW480 cells. Cells were transfected with XRCC6 siRNA, followed by overexpression of either XRCC6-WT or XRCC6-K526R, and then treated with CHX. The results demonstrated that the K526R mutation significantly enhances XRCC6 protein stability (Fig. S5C, D). We also further explored whether the XRCC6 K526R mutant affected the interaction with TRIM21. The results showed that the K526R mutation significantly reduced the interaction between XRCC6 and TRIM21(Fig. S5E, F).

Next, we explored the regulatory effect of CNOT7 on TRIM21. CNOT7 is well-known for its deadenylation function in regulating mRNA expression. Therefore, we measured the levels of TRIM21 mRNA before and after actinomycin D treatment in both CNOT7 knockdown and control cells. Compared to control cells, the stability of TRIM21 mRNA was significantly enhanced in CNOT7 knockdown cells (Fig. 6L). Additionally, CNOT7 knockdown cells showed a reduced number of P-bodies, specific cytoplasmic foci enriched with proteins involved in mRNA metabolism (Fig. 6M). Furthermore, RIP assays confirmed that CNOT7 interacts with TRIM21 mRNA (Fig. 6N). These results indicate that the deadenylation capacity is impaired in CNOT7 knockdown CRC cells, as evidenced by the reduction in P-bodies, which are associated with deadenylation activity. This impairment leads to increased stability of TRIM21 mRNA, resulting in higher expression of TRIM21 protein. The elevated TRIM21 protein promotes the degradation of XRCC6 through K48-linked polyubiquitination at the Lys526 site. Subsequently, we performed immunohistochemistry on patient tissues and found that patients insensitive to radiotherapy exhibited higher XRCC6 expression and lower TRIM21 expression compared to patients sensitive to radiotherapy (Fig. S6A, B). Immunohistochemistry also showed that XRCC6 expression was higher and TRIM21 was lower in CRC (Fig. S6C, D).

### Knockdown of XRCC6 can reverse the radiation resistance induced by CNOT7

**Fig. 7:**
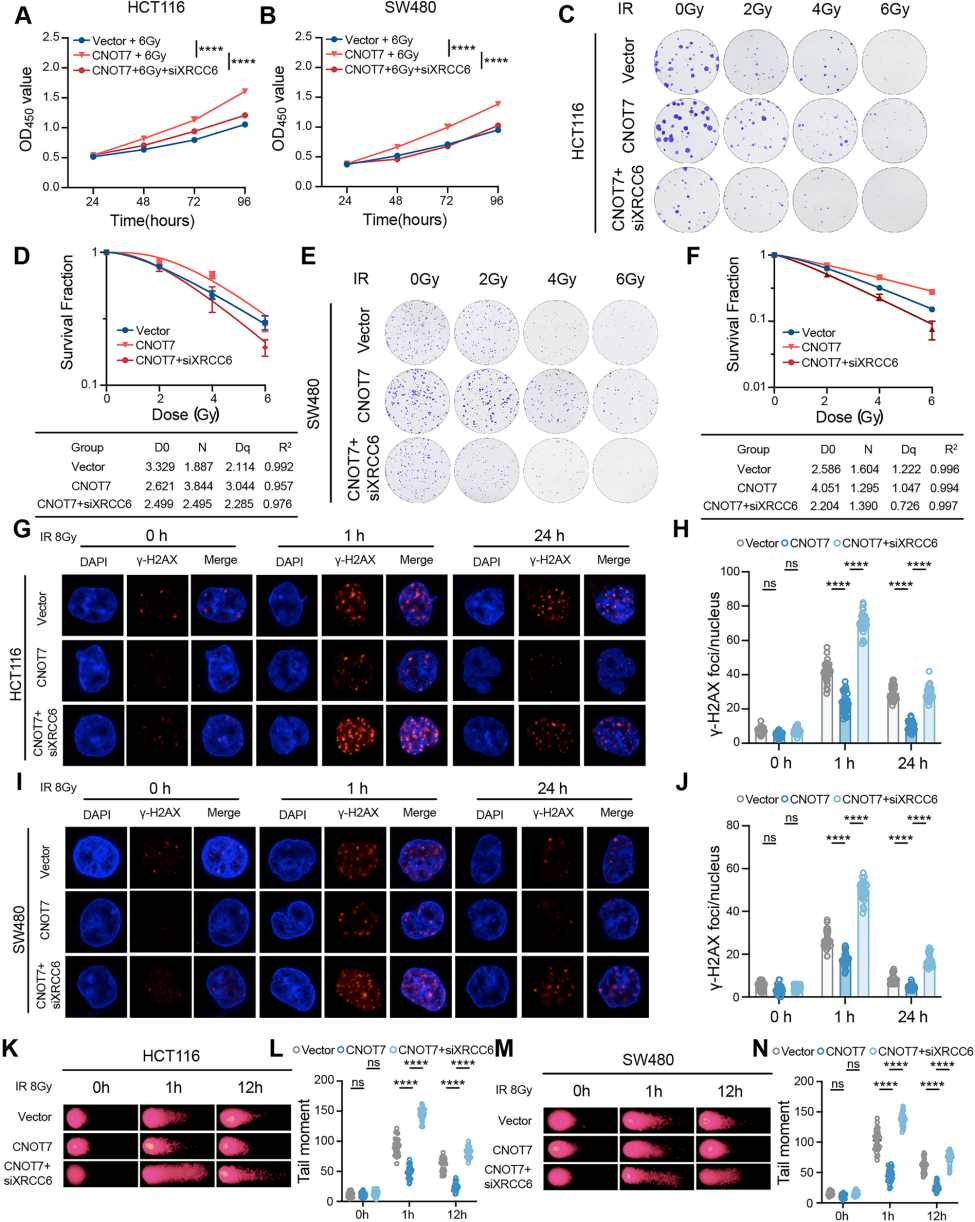
Knockdown of XRCC6 can reverse the radiation resistance induced by CNOT7. **A**, **B** CCK8 assay showed that XRCC6 knockdown increased the radiation therapy sensitivity in CNOT7 overexpression HCT116 (**A**) and SW480 (**B**) cells. **C**–**F** Representative images and corresponding survival fraction curves of colony formation assays showed that XRCC6 knockdown increased radiotherapy sensitivity in HCT116 (**C**, **D**) and SW480 cells (**E**, **F**). **G**, **H** Representative images and quantitation of γH2AX positive nuclei in HCT116, CNOT7 overexpression HCT116 cells and CNOT7 overexpression XRCC6 knockdown HCT116 cells at different time points. **I**, **J** Representative images and quantitation of γH2AX positive nuclei in SW480, CNOT7 overexpression SW480 cells and CNOT7 overexpression XRCC6 knockdown SW480 cells at different time points. **K**, **L** Representative images of comet assay and quantitative analysis of tail moment in HCT116, CNOT7 overexpression HCT116 cells and CNOT7 overexpression XRCC6 knockdown HCT116 cells at different time points. **M**, **N** Representative images of comet assay and quantitative analysis of tail moment in SW480, CNOT7 overexpression SW480 cells and CNOT7 overexpression XRCC6 knockdown SW480 cells at different time points.

Next, we further investigated whether XRCC6 knockdown could reverse radiation resistance caused by CNOT7 overexpression in CRC cells. In the CCK8 assay, we found that XRCC6 knockdown significantly reversed radiation resistance of CNOT7 overexpression cells (Fig. 7A, B). Consistent results were also obtained in colony formation assay (Fig. 7C–F). Furthermore, immunofluorescence revealed that XRCC6 knockdown significantly enhanced the accumulation of γ-H2AX in CNOT7 overexpression cells (Fig. 7G–J). Comet assay showed that XRCC6 knockdown significantly increased tail length in CNOT7 overexpression cells, a marker of DNA damage, indicating that the tumor cells experienced more severe DNA damage (Fig. 7K–N). Overall, these results suggest that the radiation resistance induced by CNOT7 overexpression in CRC cells can be reversed by the silencing of XRCC6.

### STL127705 enhances the anti-tumor effect of radiotherapy in CRC

**Fig. 8:**
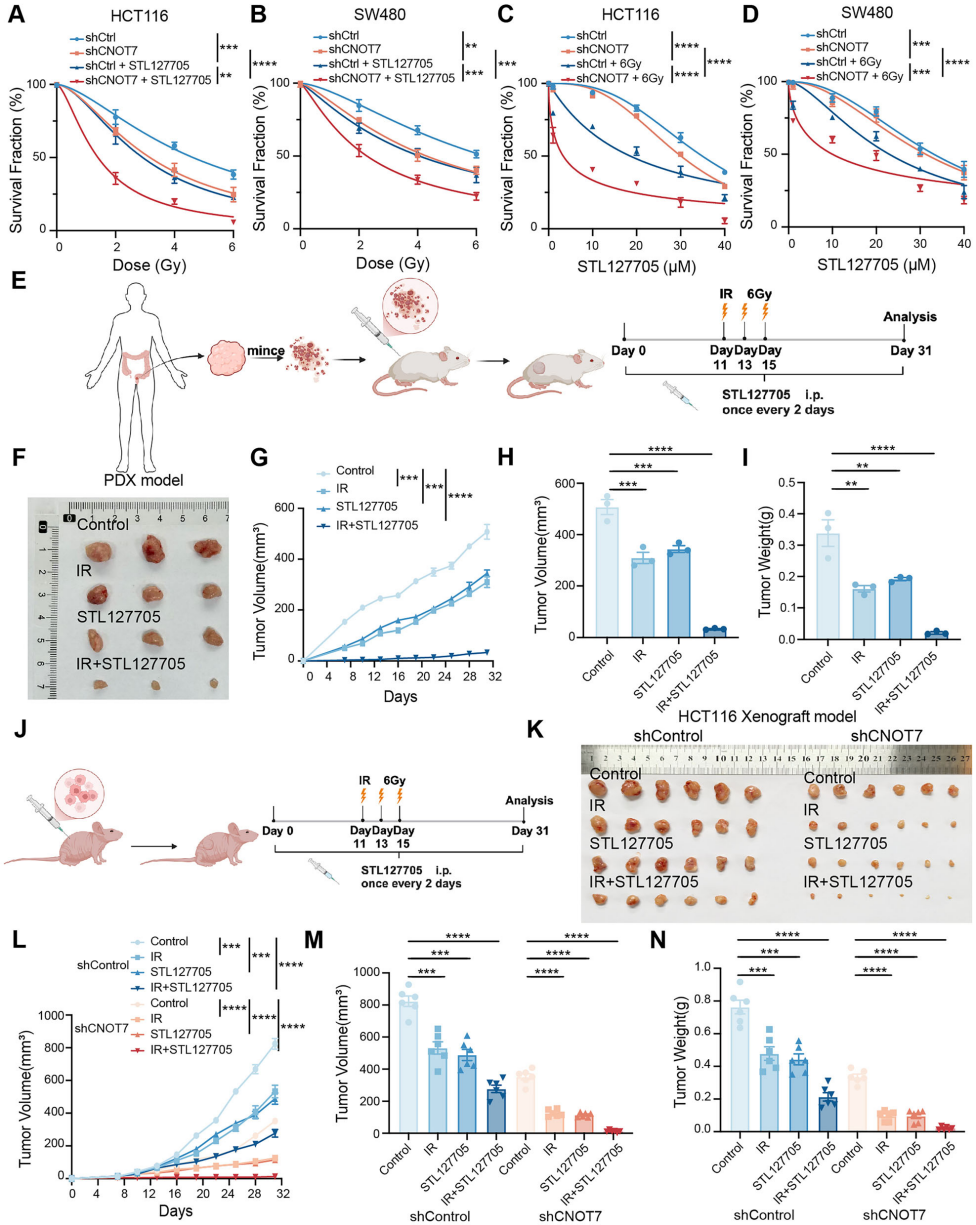
STL127705 enhances the anti-tumor effect of radiotherapy in CRC. **A** Sensitivity curves for different doses of radiation treatment in HCT116 and CNOT7 knockdown HCT116 cells with or without STL127705 (30 µM) treatment. **B** Sensitivity curves for different doses of radiation treatment in SW480 and CNOT7 knockdown SW480 cells with or without STL127705 (30 µM) treatment. **C** Sensitivity curves for different concentrations of STL127705 in HCT116 and CNOT7 knockdown HCT116 cells with or without radiation. **D** Sensitivity curves for different concentrations of STL127705 in SW480 and CNOT7 knockdown SW480 cells with or without radiation. **E** Schematic diagram of construction and drug therapy for patient-derived tumor xenograft model. **F** Gross images of patient-derived tumor xenograft with or without radiotherapy and STL127705. **G**–**I** Tumor growth curves (**G**), tumor volume (**H**) and tumor weights (**I**) of patient-derived tumor xenograft with or without radiotherapy and STL127705. **J** Schematic diagram of drug therapy for HCT116 xenograft model. **K** Gross images of HCT116 and CNOT7 knockdown HCT116 cell-derived subcutaneous tumors with or without radiotherapy and STL127705. **L**–**N** Tumor growth curves (**L**), tumor volume (**M**) and tumor weights (**N**) of HCT116 and CNOT7 knockdown HCT116 cell-derived subcutaneous tumors with or without radiotherapy and STL127705.

STL127705 has been identified as an effective inhibitor of the XRCC6/XRCC5 heterodimer protein, which can significantly suppress the DNA damage repair capacity of XRCC6. We further investigated whether the XRCC6 inhibitor increases the sensitivity of CRC to radiotherapy. Cells were subjected to varying doses of radiation and subsequently treated with STL127705. We found that CNOT7 knockdown increased the radiosensitivity of CRC and combination of STL127705 treatment significantly reduced cell viability in the CNOT7 knockdown group (Fig. 8A, B). Additionally, after receiving a fixed 6 Gy radiation dose, the combined application of STL127705 can further reduce cell viability (Fig. 8C, D). Subsequently, we used patient-derived xenograft model to further confirm the role of STL127705 combined with IR in the treatment of CRC (Fig. 8E). The tumors were irradiated locally with 6 Gy on days 11, 13, and 15 after tumor inoculation and STL127705 (50 mg/kg) was injected every two days. Our data showed combination of radiation and STL127705 notably suppressed tumor growth compared to single radiation or STL127705 treatment (Fig. 8F–I). We then validated these findings in HCT116 xenograft tumor model, and the results appeared to be consistent. Notably, this effect was more pronounced in the CNOT7 knockdown group, showing a stronger tumor-suppressive effect (Fig. 8J–N). These findings indicate that combining radiotherapy and STL127705 can significantly suppress CRC.

## Discussion

**Fig. 9:**
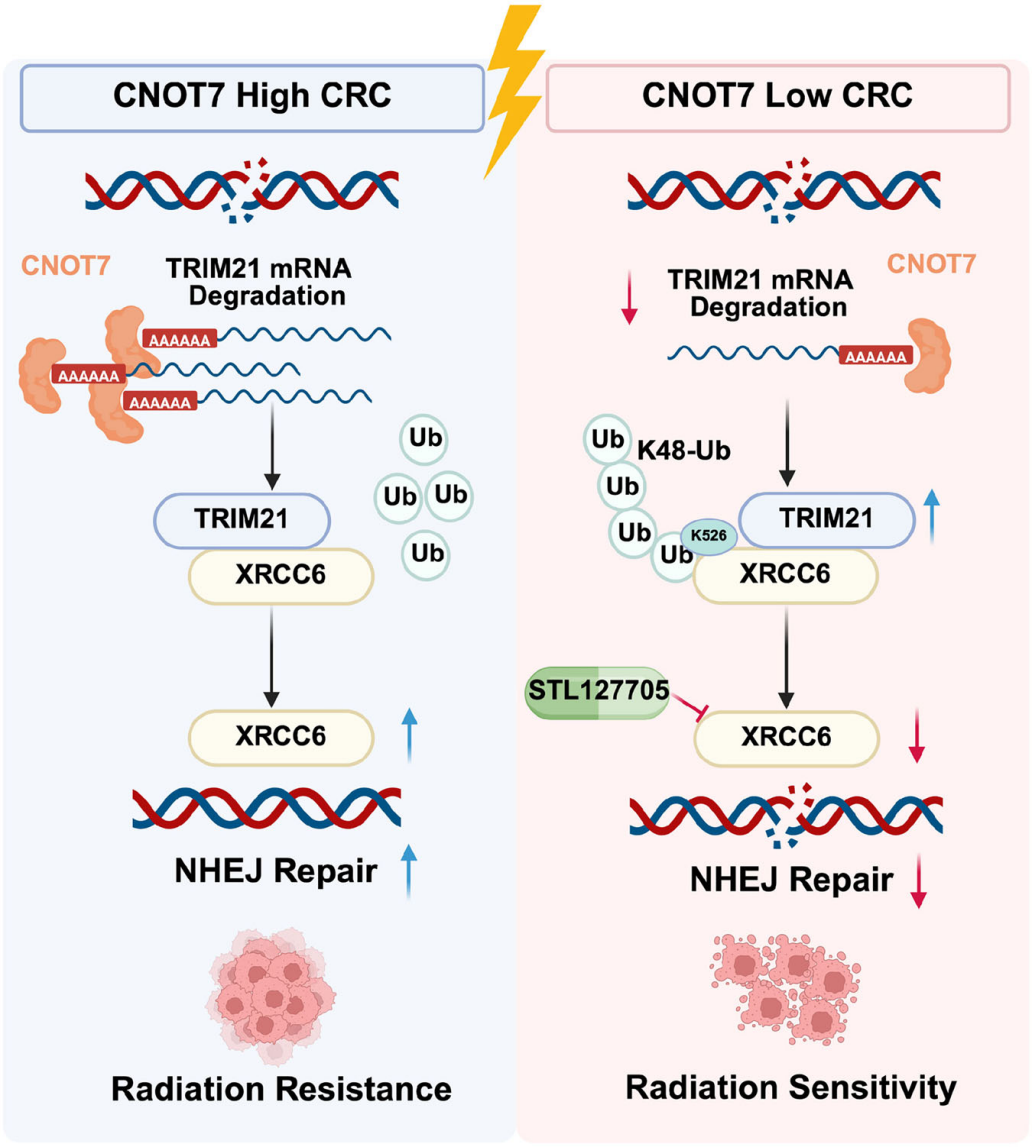
Schematic diagram of the regulatory mechanism by which CNOT7 promotes radiation resistance in colorectal cancer by regulating the XRCC6-mediated NHEJ DNA repair pathway through TRIM21.

Radiotherapy is one of the main treatment modalities for locally advanced, unresectable rectal cancer [30]. However, in some rectal cancer patients, the resistance to radiotherapy results in treatment failure and poor prognosis [4–6]. Identifying molecular targets to enhance the sensitivity of rectal cancer to radiotherapy is essential (Fig. 9). In this study, we first performed proteomic sequencing on paraffin tissues of rectal cancer without radiotherapy and grouped the samples based on the efficacy of neoadjuvant therapy to explore key genes regulating radiotherapy sensitivity of CRC. We then found that CNOT7 could serve as a target to induce radiotherapy resistance in CRC. Increasing evidence highlight CNOT7 function in tumor initiation and progression. CNOT7 and its paralog CNOT8 can influence the stability of cell cycle-related genes MSMB, PMP22, and Cyclin G2 through their deadenylation ability, promoting cell proliferation in breast cancer [31]. In glioma, CNOT7 is overexpressed and associated with poor prognosis, contributing to glioma progression through TNF-α and IL6-JAK-STAT3 pathways [10]. In osteosarcoma, CNOT7-mediated m6A methylation regulates osteosarcoma proliferation, migration, and invasion [32]. Nevertheless, there is limited research on CNOT7 in CRC, and no studies have investigated the specific mechanisms by which CNOT7 mediates radiotherapy sensitivity. In this study, we combined publicly available data and tumor samples from our center and found that CNOT7 protein is widely overexpressed in CRC tissues. Furthermore, elevated CNOT7 expression is often linked to reduced effectiveness of radiotherapy and survival analysis from databases showed that abnormal CNOT7 high- expression is closely linked to poor prognosis in stage III and IV CRC. Therefore, we constructed stable CRC cell lines with CNOT7 knockdown and overexpression to explore CNOT7 impact in vitro and in vivo. Our results showed that CNOT7 promotes invasion and migration of CRC cells. Moreover, CNOT7 knockdown markedly reduces CRC proliferation and survival after radiotherapy and enhances radiotherapy-induced apoptosis.

Radiation induces DSBs and DDR is critical for responding to radiotherapy. Efficient and accurate DNA repair contributes to genomic stability and cell survival. Studies have also shown that CNOT7’s paralog CNOT8 plays a key role in DDR [33]. Therefore, we further investigated whether CNOT7 regulates IR-induced DDR in CRC. RNA sequencing showed that differentially genes after CNOT7 knockdown were notably enriched in DNA repair pathways. Our research confirmed that CNOT7 weakened γ-H2AX accumulation and reduced the tail moment, indicating that CNOT7 plays a key role in promoting DDR. DSBs are primarily repaired through HR and NHEJ pathways. Cells with CNOT7 knockdown exhibited reduced DNA repair capacity after radiotherapy. Alterations of NHEJ-related proteins (XRCC5, XRCC6, p-DNA-Pkcs, and 53BP1) levels confirmed to this trend, while HR-related proteins such as NBS1, MRE11, and RAD51 levels did not significantly change. Gene reporter assays further demonstrated that CNOT7 primarily enhances NHEJ repair efficiency rather than affecting HR-mediated DNA damage repair, a finding not previously reported.

To explore the mechanism by which CNOT7 influences NHEJ repair in CRC cells, we conducted immunoprecipitation (IP) and mass spectrometry (MS) and identified that the NHEJ downstream key molecule XRCC6 interacts with CNOT7. XRCC6 is a critical downstream molecule of NHEJ and can interact with XRCC5 to bind DNA DSB ends, protecting the broken DNA and activating downstream DNA-Pkcs phosphorylation. Elevated XRCC6 is associated with poor prognosis in multiple cancers and significantly affects the sensitivity of tumors to radiotherapy [34–36]. XRCC6 undergoes post-translational modifications like methylation, palmitoylation, and acetylation to affect cell proliferation and DDR [37–40]. Here, we discovered that CNOT7 interacts with XRCC6 and suppresses its degradation through ubiquitin-proteasome pathway. Since CNOT7 is not key enzyme for influencing ubiquitination, we hypothesize that a molecular factor responsible for affecting XRCC6 ubiquitination might exist between CNOT7 and XRCC6. Through IP-MS analysis, we identified TRIM21. TRIM family members are essential for many processes, such as cell proliferation, differentiation, maintaining tumor stemness, and DNA damage repair [41–43]. Our study showed that TRIM21 interacts with XRCC6. The ubiquitination activity of TRIM21 directly influences XRCC6 protein stability. We also found that TRIM21 promotes XRCC6 degradation through K48-linked polyubiquitination, with Lys526 being a key site for TRIM21-mediated ubiquitination. Meanwhile, CNOT7, a key deadenylase subunit, mediates poly(A) tail removal to accelerate TRIM21 mRNA degradation and reduces TRIM21 expression, further enhancing the protein stability of XRCC6. Previous studies have demonstrated that CNOT7 promotes tumor proliferation and progression. In this study, we report for the first time a specific regulatory role of CNOT7 in NHEJ, adding an important dimension to the intricate regulatory network of DNA repair. DNA repair pathways are tightly orchestrated by post-translational modifications (PTMs), transcriptional regulation, and protein–protein interactions. Stabilization of key repair factors is a well-established strategy to enhance repair capacity. For example, deubiquitinating enzymes (DUBs) such as USP9X stabilize BRCA1 to promote HR, whereas acetyltransferases like TIP60 modify ATM and histone H4 to facilitate DDR signaling [44–46]. Our findings identify a novel regulatory axis in which CNOT7, an mRNA deadenylase, modulates NHEJ not by direct enzymatic modification of DNA repair proteins, but by controlling the stability of the key NHEJ factor XRCC6 through regulation of its ubiquitin ligase, TRIM21. Unlike canonical DDR kinases (ATM, ATR, DNA-PK) or scaffold proteins (53BP1, BRCA1), CNOT7 underscores the significance of mRNA stability as an underappreciated layer of DDR regulation—particularly in modulating NHEJ within the context of radioresistance. Moreover, CNOT7 overexpression significantly increased CRC cells resistance to radiotherapy, while XRCC6 knockdown reversed this effect. In CRC xenograft model, CNOT7 deficiency significantly inhibited tumor growth and made these tumors more sensitive to radiotherapy-induced DNA damage. STL127705 (Compound L), a potent inhibitor of the XRCC6/XRCC5 heterodimer [47], can eliminate the binding of XRCC6 complex. It has been demonstrated to suppress proliferation and increase radiotherapy sensitivity [48–50]. Our results in xenograft and PDX models further revealed that combining IR with STL127705 resulted in an efficient anti-tumor effect, providing valuable reference for future clinical translation.

While our study delineates a novel CNOT7-TRIM21-XRCC6 axis in regulating NHEJ and radioresistance in CRC, several limitations warrant consideration. Firstly, we mainly studied the regulatory effect of CNOT7 on the NHEJ pathway. Although the HR protein was evaluated, a more comprehensive analysis was not conducted on other DSB repair pathways affected by CNOT7 (such as alternative terminal junctions). Secondly, this study was mainly conducted in CRC, and the correlation between CNOT7 and radiosensitivity can be further discussed in multiple tumor types. Finally, although STL127705 has certain therapeutic effects, its specificity in vivo, potential off-target effects, pharmacokinetics and toxicity need to be fully evaluated before clinical trials.

## Conclusions

In conclusion, our study shows that elevated CNOT7 expression is strongly linked to radiotherapy resistance and poor prognosis in CRC. Mechanistically, CNOT7 regulates ubiquitination of XRCC6 K48 chain and Lys526 site through TRIM21, thereby stabilizing XRCC6 protein. XRCC6 promote DNA damage repair via the NHEJ pathway, imparting radiotherapy resistance to CRC cells. Our study indicates that CNOT7 could serve a potential therapeutic target to overcome radiotherapy resistance in CRC.

## Supplementary information

Supplementary Figure and Figure Legends

Supplementary table 1. Clinical information

Supplementary table 2. Raw data of IP-MS

WB Raw Data

## Data Availability

The data generated in this study are publicly available in the Gene Expression Omnibus (GEO) under accession number “GSE287014” (RNA-Seq). The raw data of IP-MS can be found in Supplementary Table 2. All other raw data are available upon request from the corresponding author.
