# Supplementary material for: CNOT7 facilitates radiation resistance in colorectal cancer through TRIM21/XRCC6-mediated non-homologous end joining repair
Source: Cell Death Dis. 2025 Nov 17;16(1):833. doi: 10.1038/s41419-025-08160-4 (PMC12623751; doi:10.1038/s41419-025-08160-4)
Supplement: Supplementary file 1 — Supplementary Figure and Figure Legends [file 41419_2025_8160_MOESM1_ESM.pdf]

Supplementary Figure and Figure Legends

Supplementary Figure 1. Expression levels of CNOT7 in tumor tissues and adjacent normal tissues in various cancers.

(A): Statistical analysis of CNOT7 IHC staining score between CR, PR and SD group. (B): Representative immunohistochemical images of CNOT7 in CRC and adjacent normal tissues. Scale bar, 60 μm. (C-H): The mRNA expression levels of CNOT7 in tumor tissues and adjacent normal tissues in the GEPIA database. (I-J): The mRNA (I) and protein (J) expression levels of CNOT7 in multiple CRC cell lines.

Supplementary Figure 2. Knockdown of CNOT7 inhibits cell migration and invasion.

(A-D): Validation of CNOT7 knockdown (A-B) and overexpression (C-D) at the protein level in HCT116 and SW480 cells. (E-H): The mRNA level of CNOT7 in WT, CNOT7 knockdown and CNOT7 overexpression cells. (I-J): The wound healing assay showed that knockdown of CNOT7 inhibits cell migration. (K-M): The transwell assay showed that knockdown of CNOT7 inhibits cell migration and invasion.

Supplementary Figure 3. knockdown of CNOT7 promotes sensitivity of CRC to radiotherapy in vitro.

(A-B): Representative images and corresponding survival fraction curves of colony formation assays showed that CNOT7 knockdown increased radiotherapy sensitivity in SW480. (C-D): Representative images and corresponding survival fraction curves of colony formation assays showed that overexpression of CNOT7 promotes radiation resistance in HCT116. (E): The bar chart showed the apoptosis ratio of SW480 and CNOT7 knockdown SW480 cells with or without radiotherapy.

Supplementary Figure 4. CNOT7 knockdown leads to accumulation of γH2AX after radiotherapy.

(A-B): Representative images and quantitation of γ-H2AX positive nuclei in SW480 and CNOT7 knockdown SW480 cells at different time points. (C-D): Representative images and quantitation of γH2AX positive nuclei in HCT116 and CNOT7 overexpression HCT116 cells at different time points. (E-F): Representative images of the comet assay and quantitative analysis of tail moment of SW480 and CNOT7 knockdown SW480 cells at different time points. (G-H)

Representative images of the comet assay and quantitative analysis of tail moment of HCT116 and CNOT7 overexpression HCT116 cells at different time points.

Supplementary Figure 5. CNOT7 recruits TRIM21 to deubiquitylate and stabilize XRCC6.

(A): Western blotting of CNOT7, XRCC6 and TRIM21 in SW480 and CNOT7 knockdown SW480 cells. (B): Western blotting of CNOT7, XRCC6 and TRIM21 in SW480 and TRIM21 knockdown SW480 cells. (C): Western blotting of XRCC6 expression in HCT116 cells transfected with siXRCC6 and XRCC6-WT or XRCC6-K526R plasmids following cycloheximide (CHX 100μg/ml). (D): Western blotting of XRCC6 expression in SW480 cells transfected with siXRCC6 and XRCC6-WT or XRCC6-K526R plasmids following cycloheximide (CHX 100μg/ml). (E): Co-IP analysis of TRIM21-XRCC6 interaction in HCT116 cells transfected with XRCC6-WT or XRCC6-K526R plasmids. Lysates immunoprecipitated with IgG and anti-TRIM21 antibody. Immunoblots probed with Anti-His and Anti-TRIM21 antibodies. (F): Co-IP analysis of TRIM21-XRCC6 interaction in SW480 cells transfected with XRCC6-WT or XRCC6-K526R plasmids. Lysates immunoprecipitated with IgG and anti-TRIM21 antibody. Immunoblots probed with Anti-His and Anti-TRIM21 antibodies.

Supplementary Figure 6. Expression levels of XRCC6 and TRIM21 in tumor tissues and adjacent normal tissues.

(A-B): Representative immunohistochemical images of XRCC6 (A) and TRIM21 (B) in different treatment groups. Right scale bar, 60 μm. (C-D): Representative immunohistochemical images of XRCC6 (C) and TRIM21 (D) in CRC and adjacent normal tissues. Scale bar, 60 μm.

Figure S1

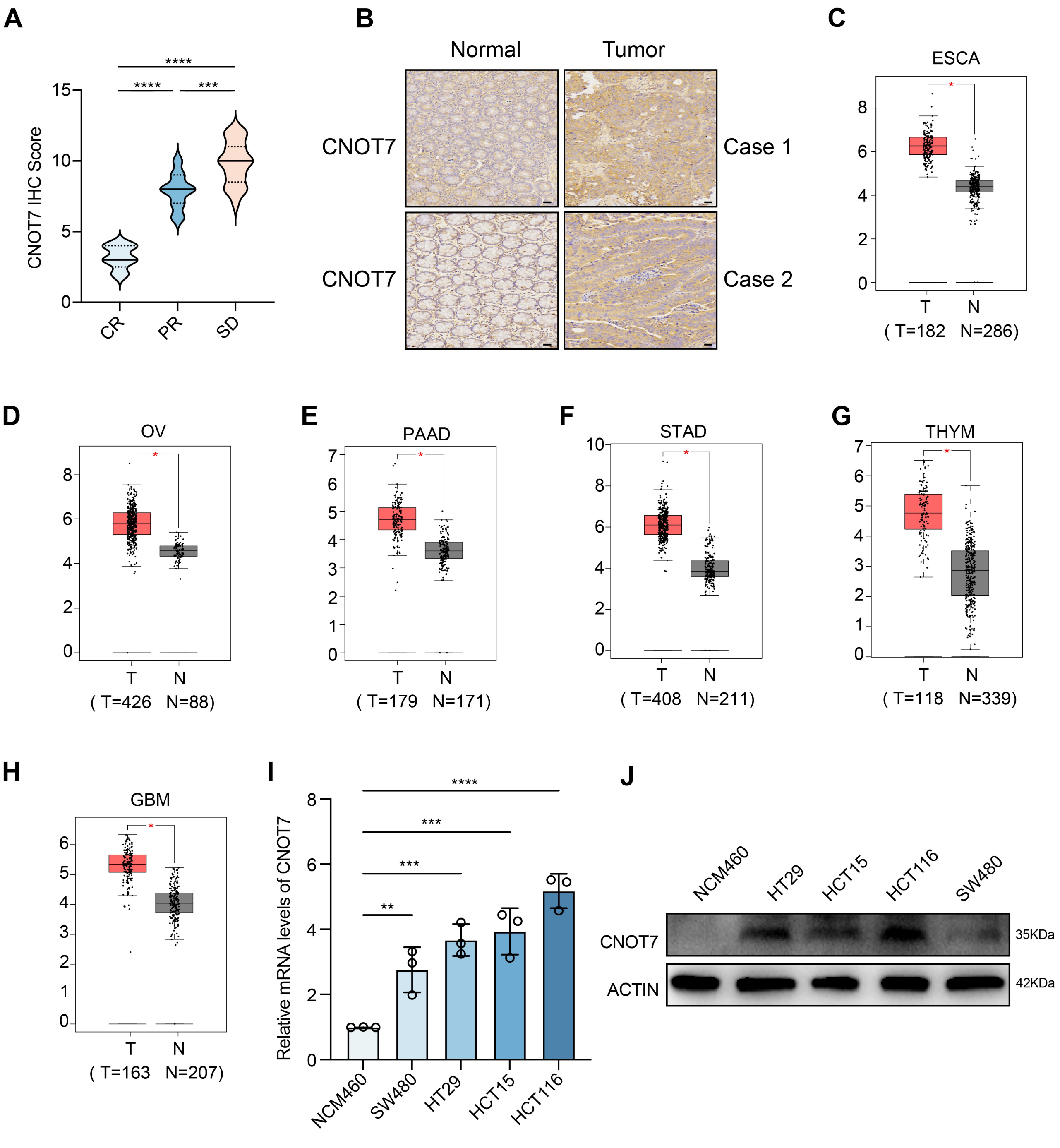

A
CNOT7 IHC Score
CR
PR
SD
B
Normal
Tumor
CNOT7
Case 1
Case 2
C
ESCA
(T=182 N=286)
D
OV
(T=426 N=88)
E
PAAD
(T=179 N=171)
F
STAD
(T=408 N=211)
G
THYM
(T=118 N=339)
H
GBM
(T=163 N=207)
I
Relative mRNA levels of CNOT7
NCM460
SW480
HT29
HCT15
HCT116
J
CNOT7
ACTIN
35KDa
42KDa

Figure S2

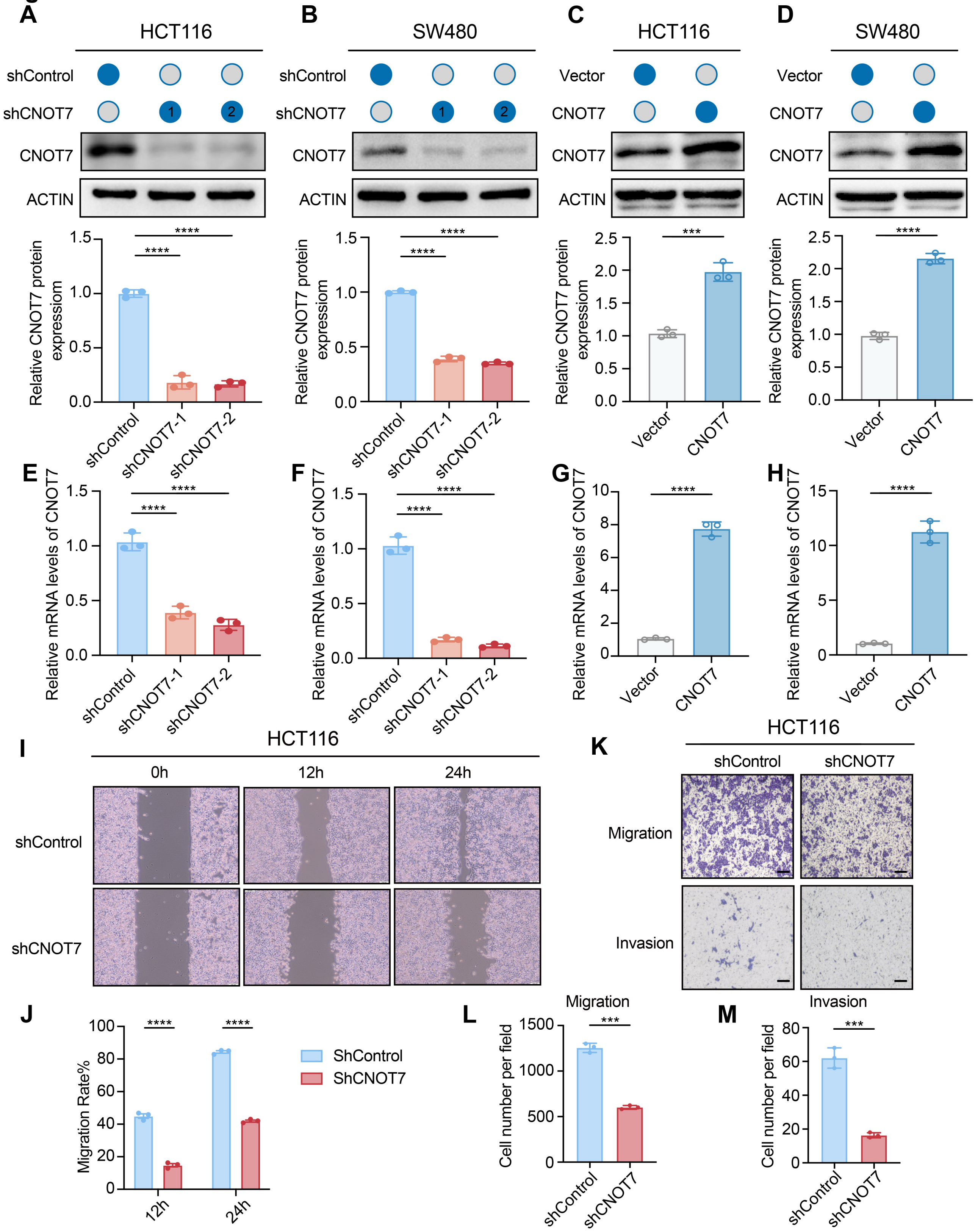
A
HCT116
shControl
shCNOT7
CNOT7
ACTIN
Relative CNOT7 protein expressiom
shControl
shCNOT7-1
shCNOT7-2
B
SW480
C
HCT116
Vector
CNOT7
D
SW480
E
Relative mRNA levels of CNOT7
F
G
H
I
HCT116
0h
12h
24h
shControl
shCNOT7
J
Migration Rate%
ShControl
ShCNOT7
12h
24h
K
HCT116
Migration
Invasion
L
Migration
Cell number per field
M
Invasion

Figure S3

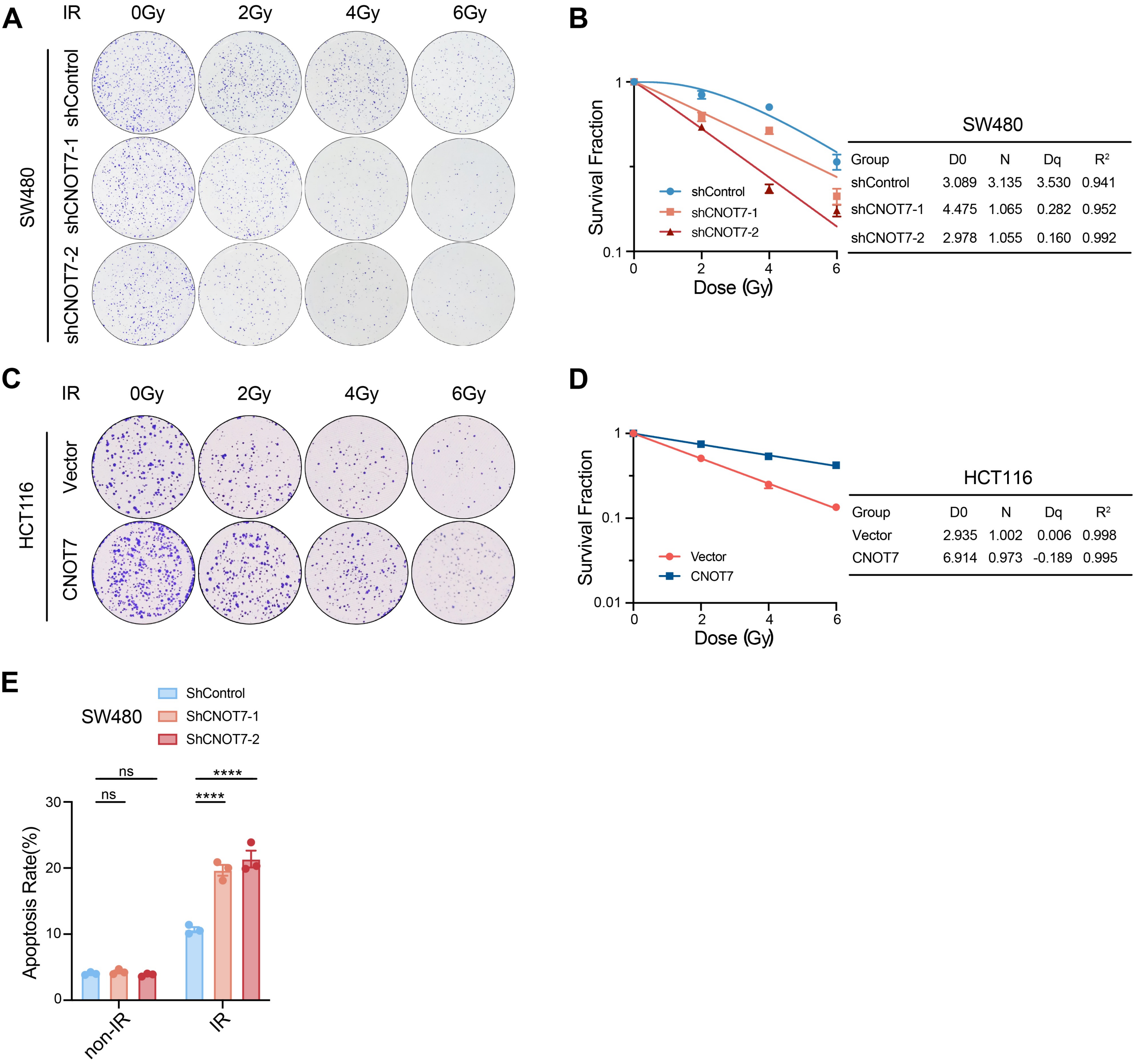
A
IR
0Gy
2Gy
4Gy
6Gy
SW480
shControl
shCNOT7-1
shCNOT7-2
B
Survival Fraction
Dose (Gy)
shControl
shCNOT7-1
shCNOT7-2
SW480
Group D0 N Dq R²
shControl 3.089 3.135 3.530 0.941
shCNOT7-1 4.475 1.065 0.282 0.952
shCNOT7-2 2.978 1.055 0.160 0.992
C
IR
0Gy
2Gy
4Gy
6Gy
HCT116
Vector
CNOT7
D
Survival Fraction
Dose (Gy)
Vector
CNOT7
HCT116
Group D0 N Dq R²
Vector 2.935 1.002 0.006 0.998
CNOT7 6.914 0.973 -0.189 0.995
E
SW480
ShControl
ShCNOT7-1
ShCNOT7-2
ns
ns
****
****
Apoptosis Rate(%)
non-IR
IR

Figure S4

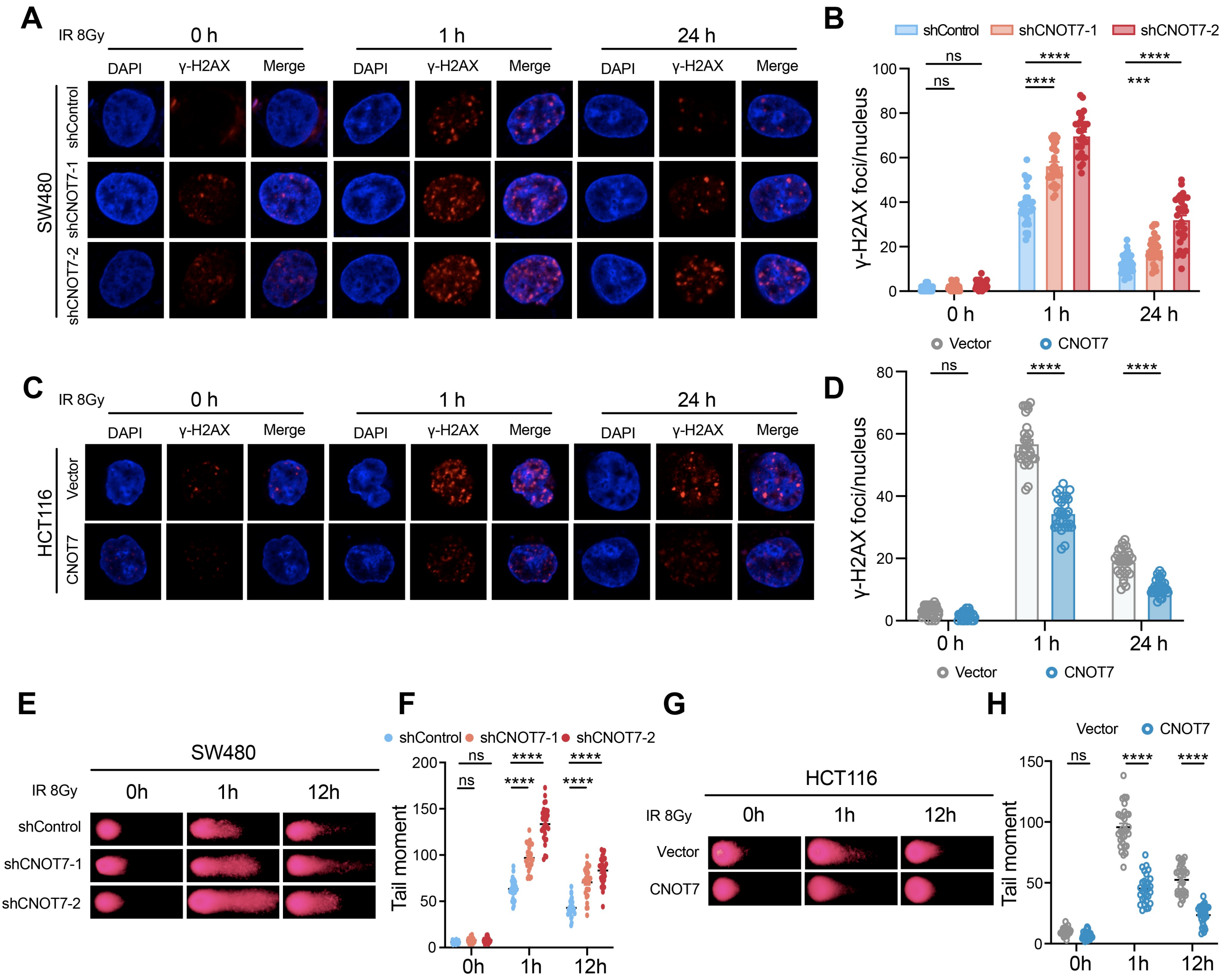

A
IR 8Gy
0 h
1 h
24 h
DAPI
γ-H2AX
Merge
SW480
shControl
shCNOT7-1
shCNOT7-2
B
shControl
shCNOT7-1
shCNOT7-2
γ-H2AX foci/nucleus
ns
****
***
C
HCT116
Vector
CNOT7
D
E
SW480
0h
1h
12h
F
Tail moment
G
HCT116
H

Figure S5

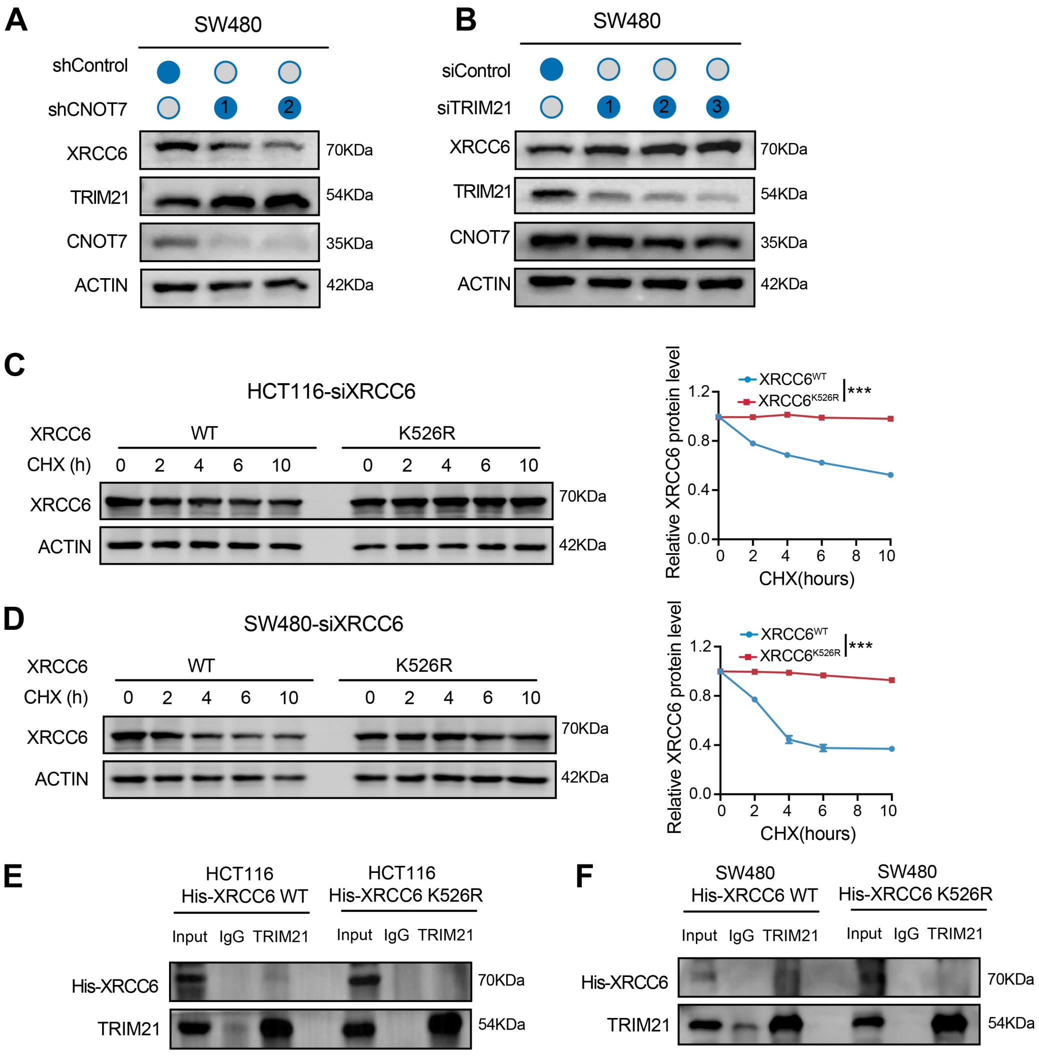
A
SW480
shControl
shCNOT7
1
2
XRCC6
70KDa
TRIM21
54KDa
CNOT7
35KDa
ACTIN
42KDa
B
SW480
siControl
siTRIM21
1
2
3
XRCC6
70KDa
TRIM21
54KDa
CNOT7
35KDa
ACTIN
42KDa
C
HCT116-siXRCC6
XRCC6
WT
K526R
CHX (h)
0 2 4 6 10
0 2 4 6 10
XRCC6
70KDa
ACTIN
42KDa
XRCC6^WT
XRCC6^K526R
***
Relative XRCC6 protein level
1.2
0.8
0.4
0.0
0 2 4 6 8 10
CHX(hours)
D
SW480-siXRCC6
XRCC6
WT
K526R
CHX (h)
0 2 4 6 10
0 2 4 6 10
XRCC6
70KDa
ACTIN
42KDa
XRCC6^WT
XRCC6^K526R
***
Relative XRCC6 protein level
1.2
0.8
0.4
0.0
0 2 4 6 8 10
CHX(hours)
E
HCT116
His-XRCC6 WT
HCT116
His-XRCC6 K526R
Input IgG TRIM21
Input IgG TRIM21
His-XRCC6
70KDa
TRIM21
54KDa
F
SW480
His-XRCC6 WT
SW480
His-XRCC6 K526R
Input IgG TRIM21
Input IgG TRIM21
His-XRCC6
70KDa
TRIM21
54KDa

Figure S6

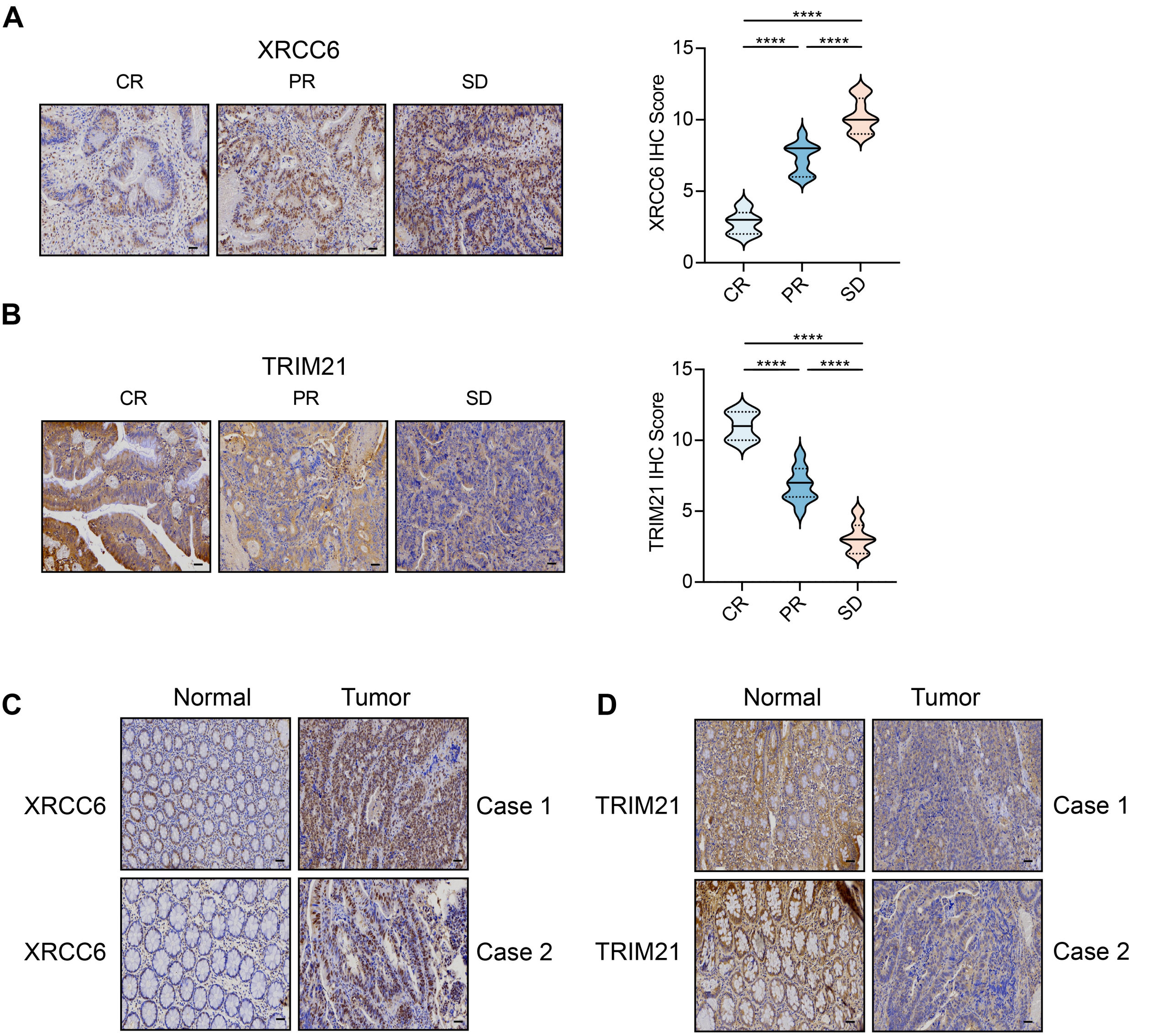
A
XRCC6
CR
PR
SD
****
****
****
15
10
5
0
XRCC6 IHC Score
CR
PR
SD
B
TRIM21
CR
PR
SD
****
****
****
15
10
5
0
TRIM21 IHC Score
CR
PR
SD
C
Normal
Tumor
XRCC6
Case 1
XRCC6
Case 2
D
Normal
Tumor
TRIM21
Case 1
TRIM21
Case 2
