# Supplementary material for: CNOT7 facilitates radiation resistance in colorectal cancer through TRIM21/XRCC6-mediated non-homologous end joining repair
Source: Cell Death Dis. 2025 Nov 17;16(1):833. doi: 10.1038/s41419-025-08160-4 (PMC12623751; doi:10.1038/s41419-025-08160-4)

Figure 1I

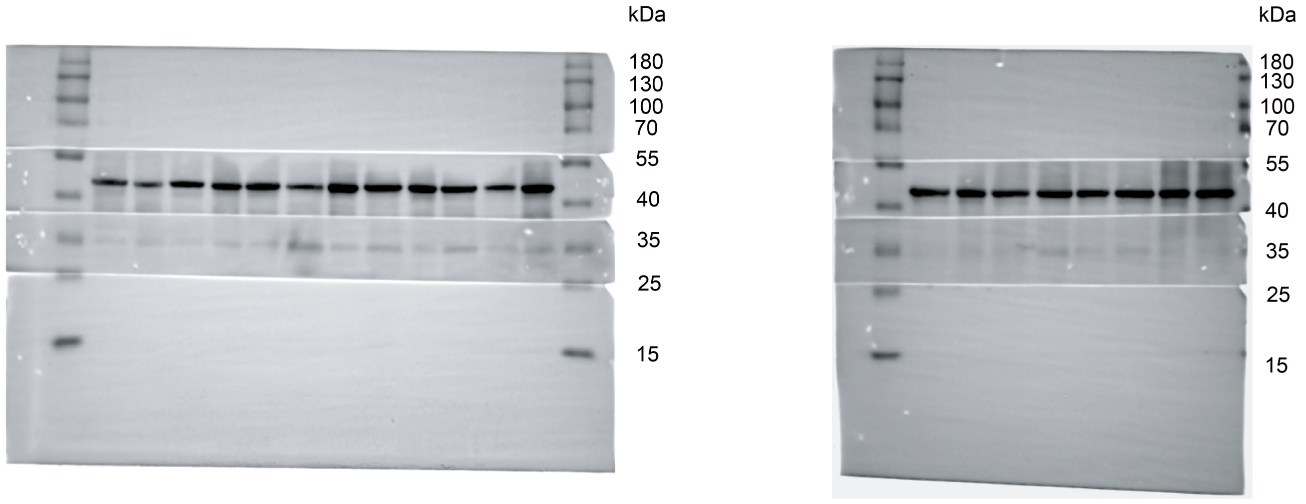

Figure 3H

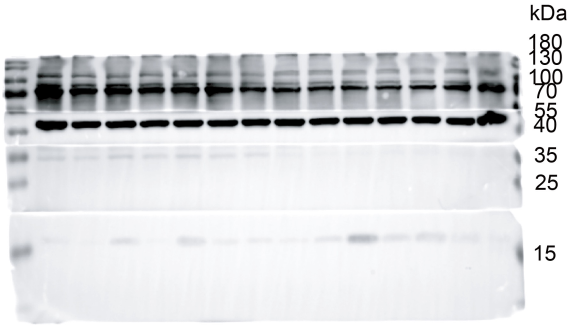

Figure 3I

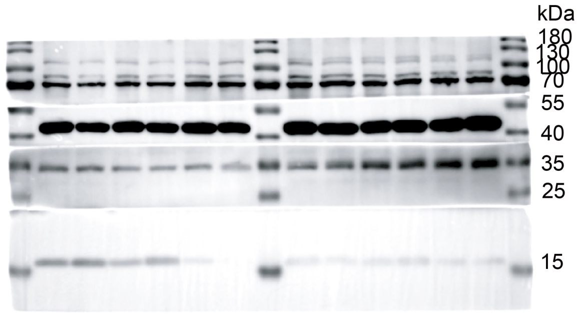

Figure 4A

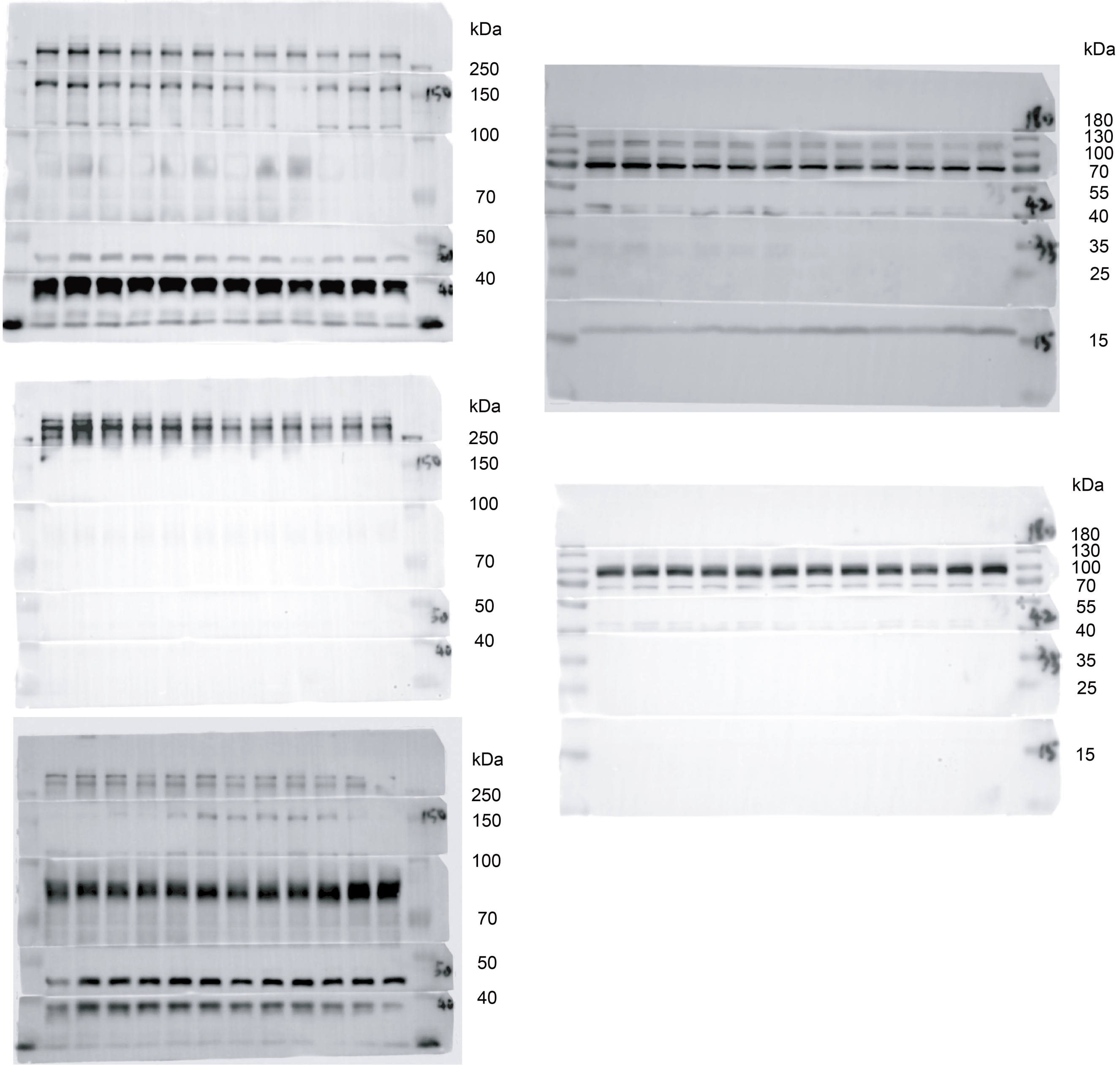
kDa
250
150
100
70
50
40
kDa
250
150
100
70
50
40
kDa
250
150
100
70
50
40
kDa
180
130
100
70
55
40
35
25
15
kDa
180
130
100
70
55
40
35
25
15

Figure 4B

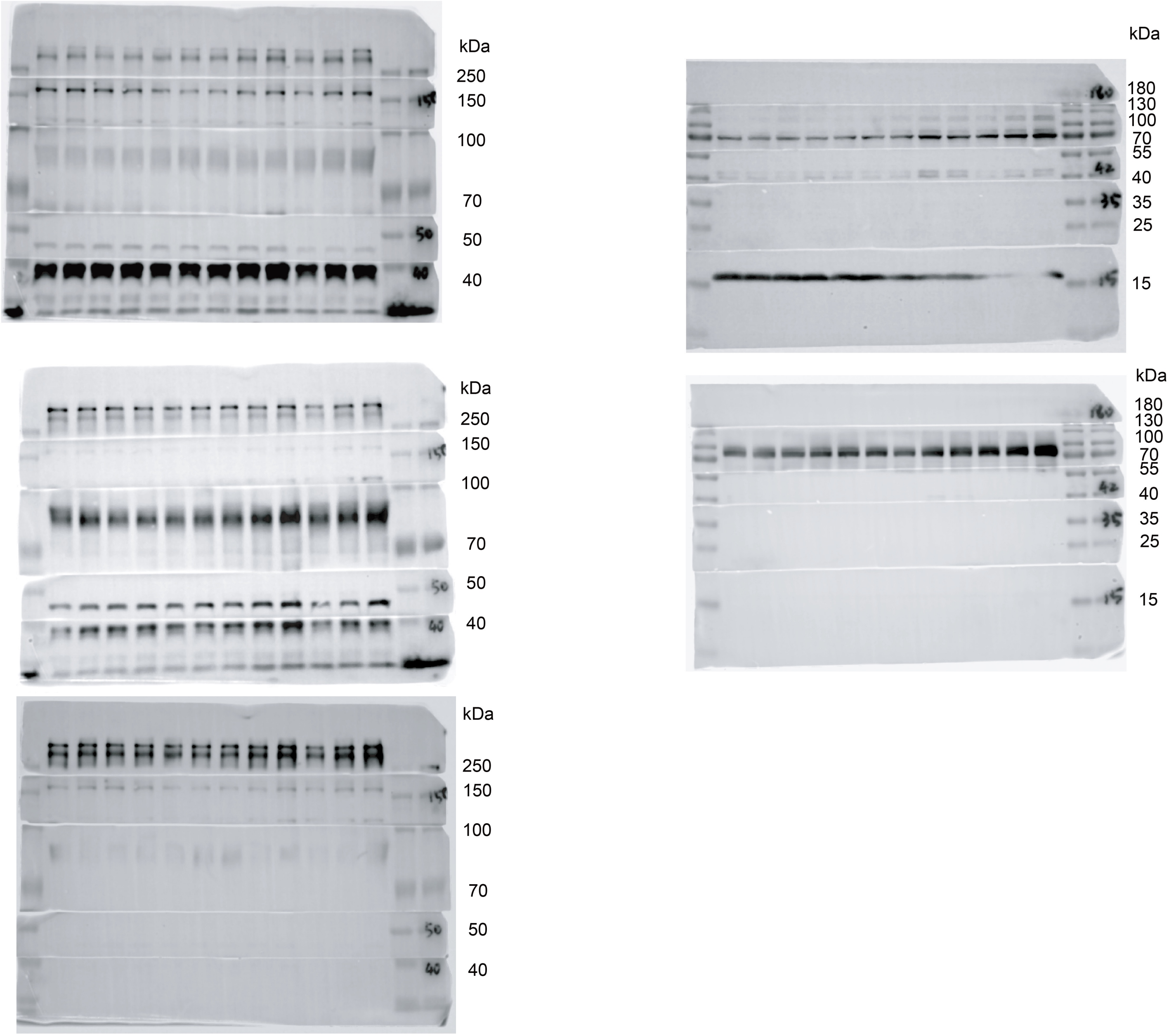
kDa
250
150
100
70
50
40
kDa
250
150
100
70
50
40
kDa
250
150
100
70
50
40
kDa
180
130
100
70
55
40
35
25
15
kDa
180
130
100
70
55
40
35
25
15

Figure 5B

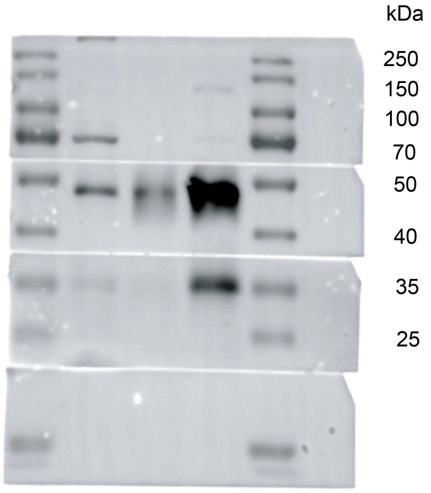

Figure 5C

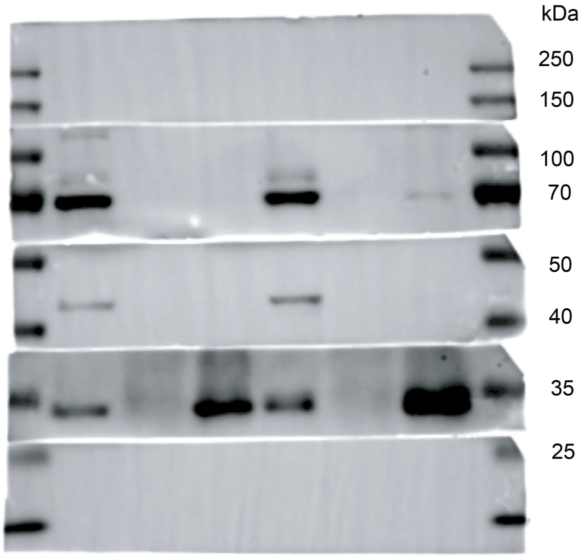

Figure 5D

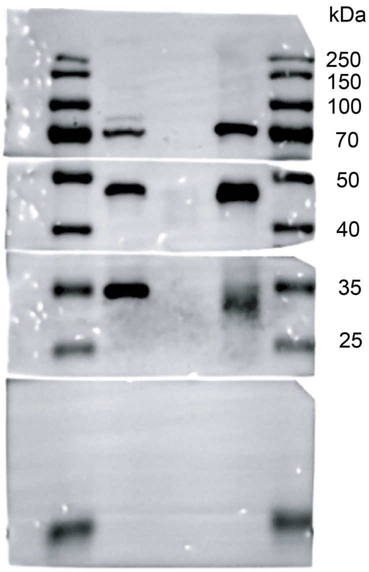

Figure 5E

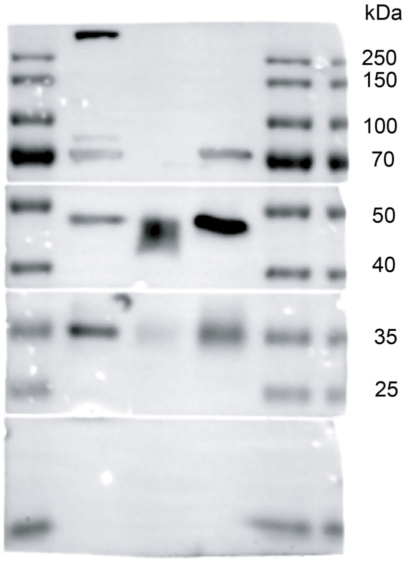

Figure 5F

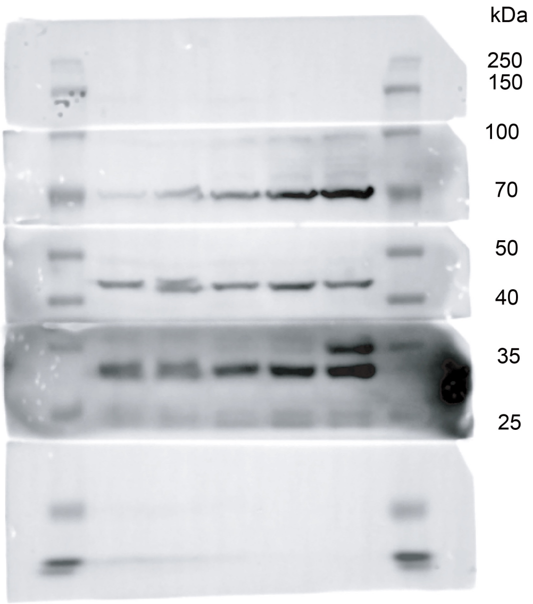

Figure 5G

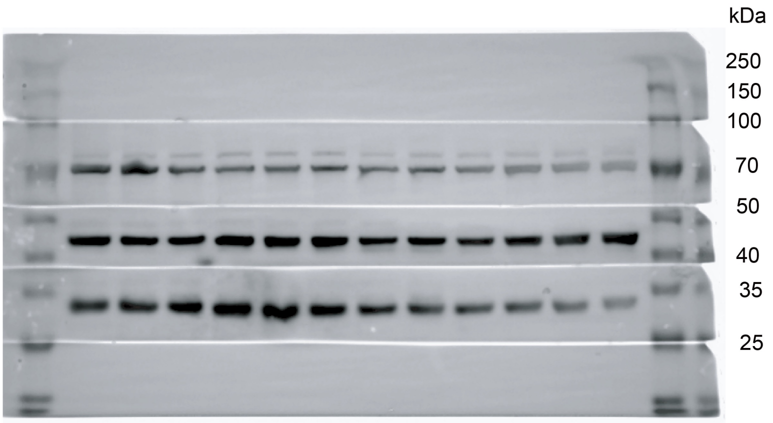

Figure 5H

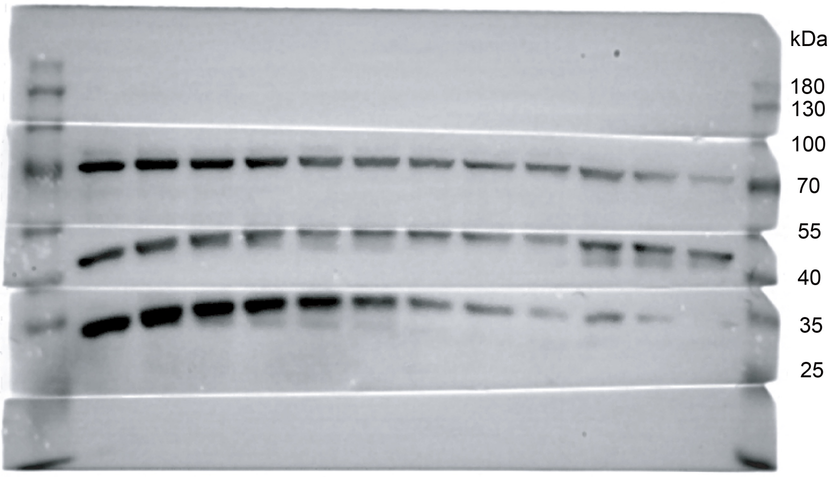

Figure 5I

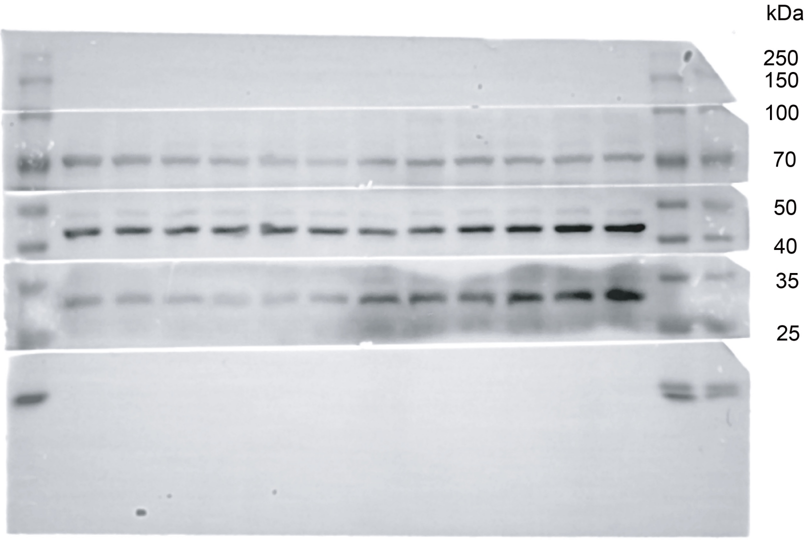

Figure 5J

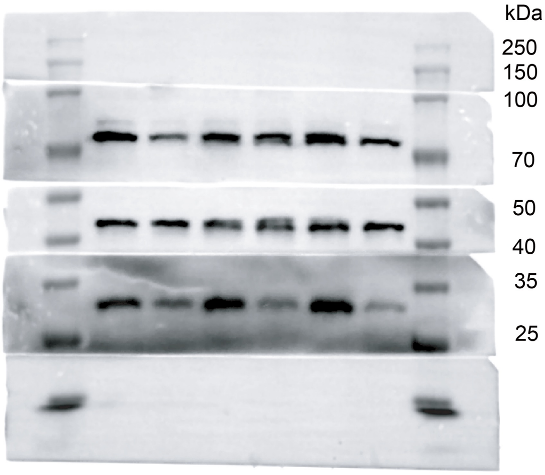

Figure 5K

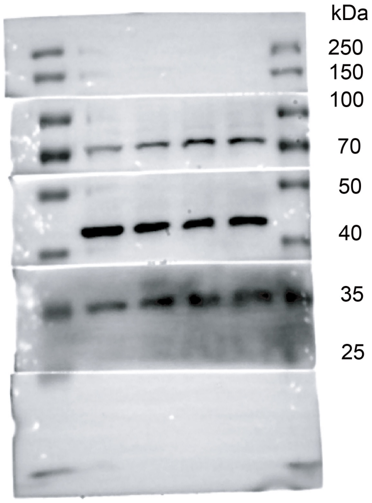

Figure 5L

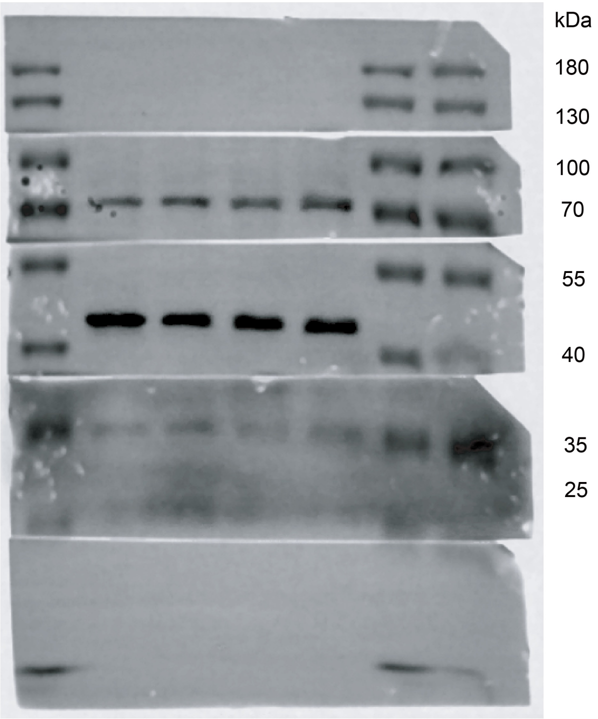

Figure 5M

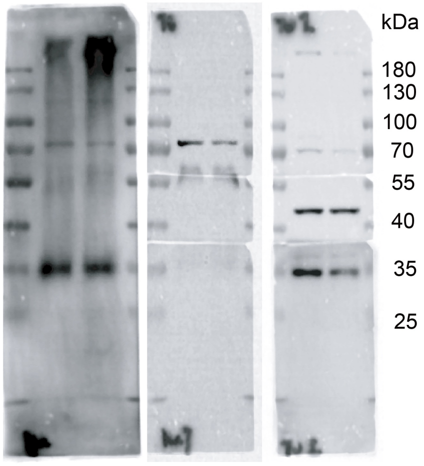

Figure 5N

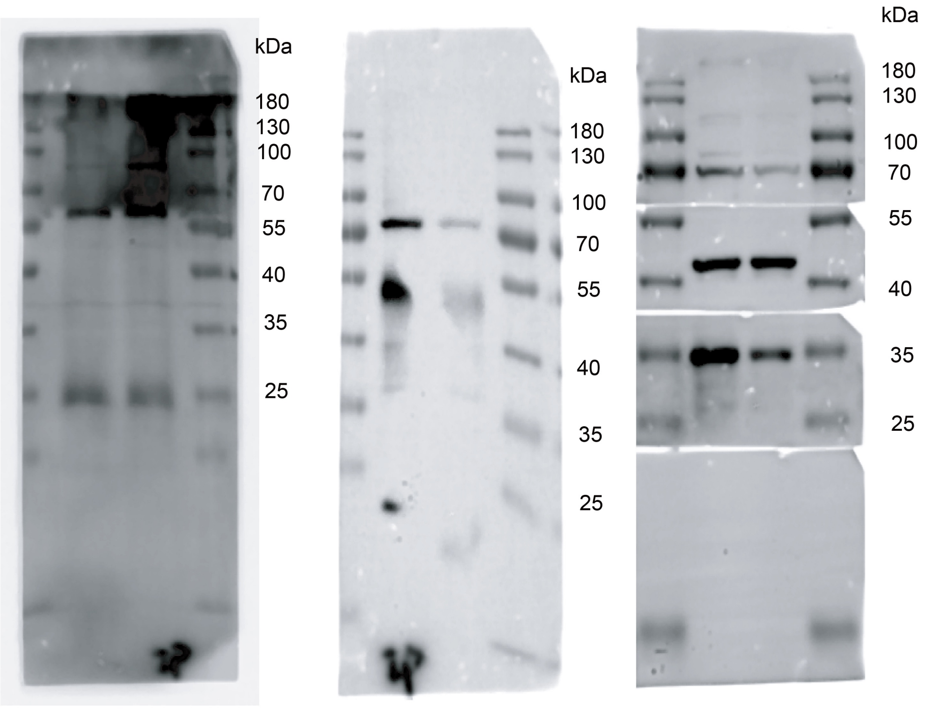

Figure 5O

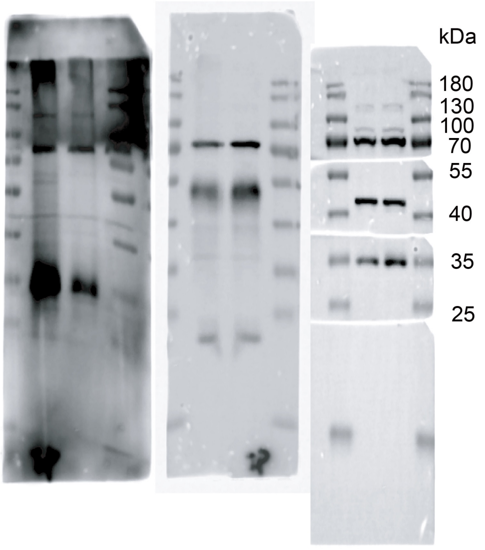

Figure 5P

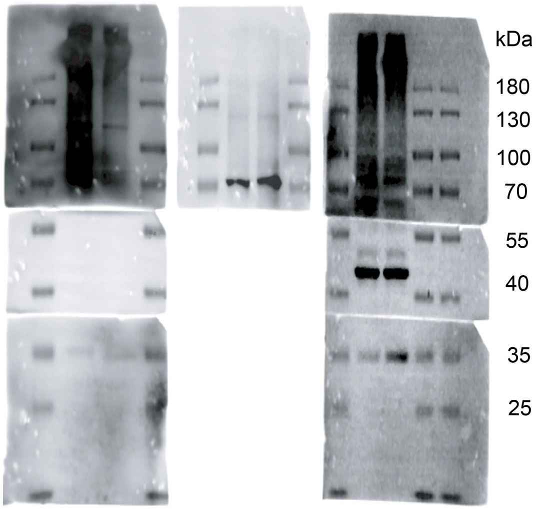

Figure 6F

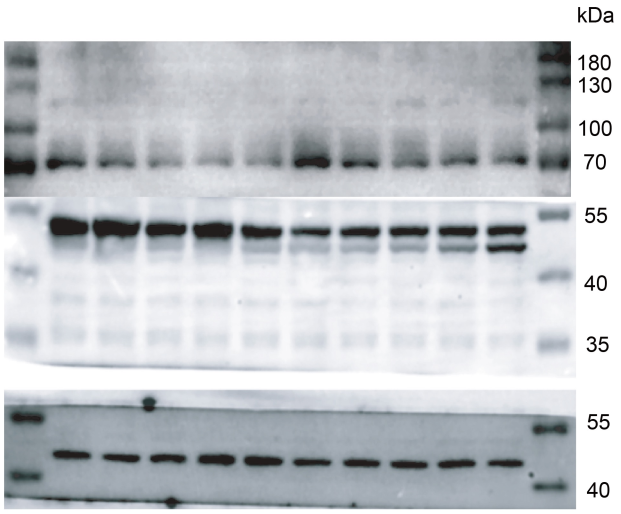

Figure 6G

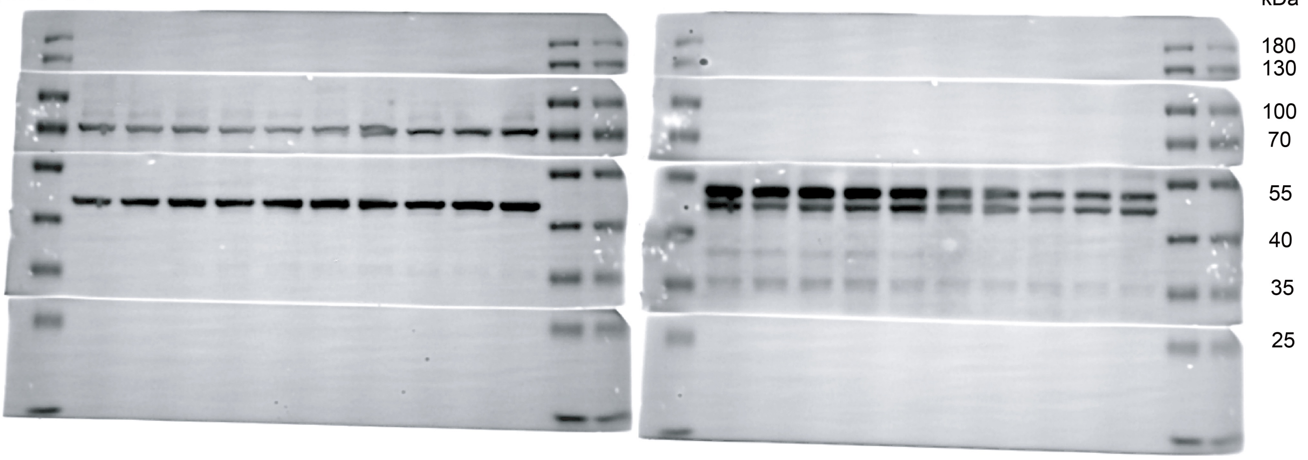

Figure 6H

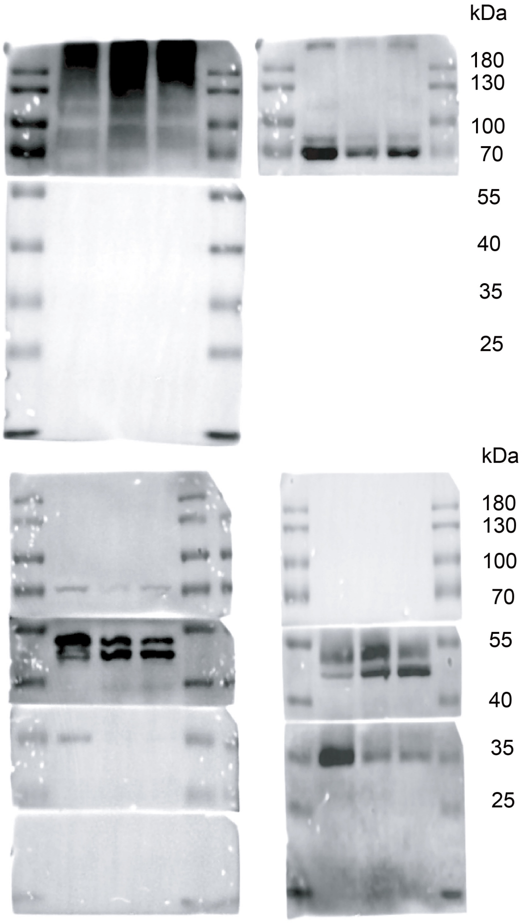

Figure 6I

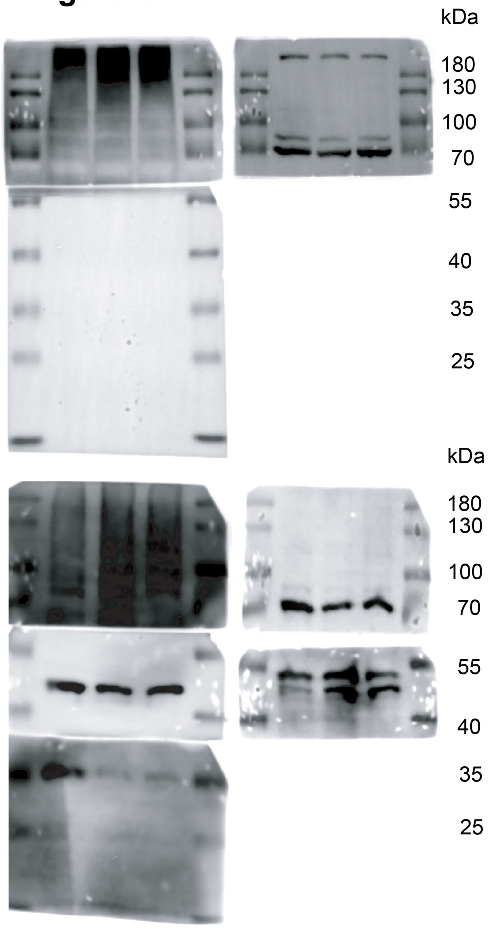

Figure 6J

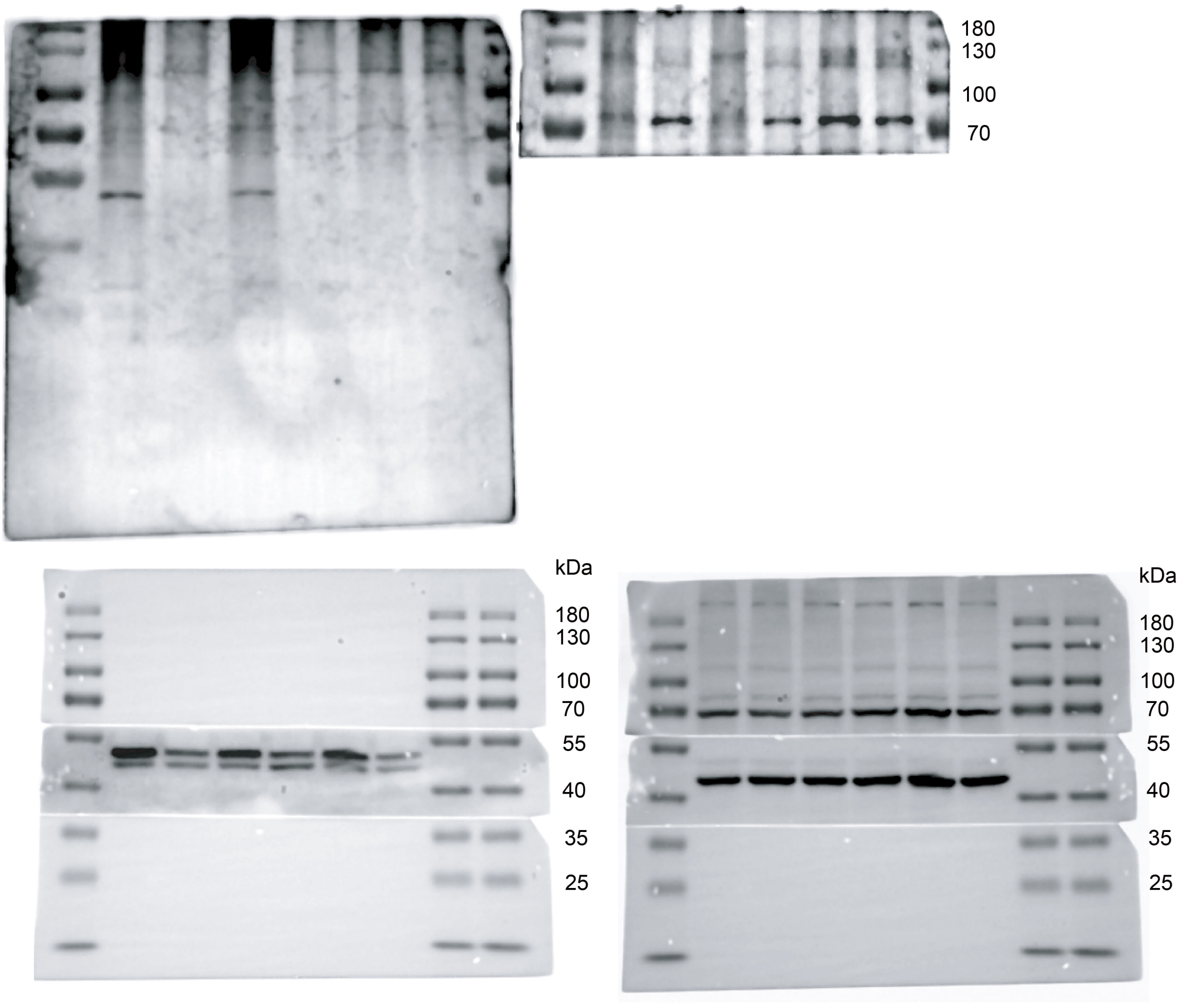

Figure 6K

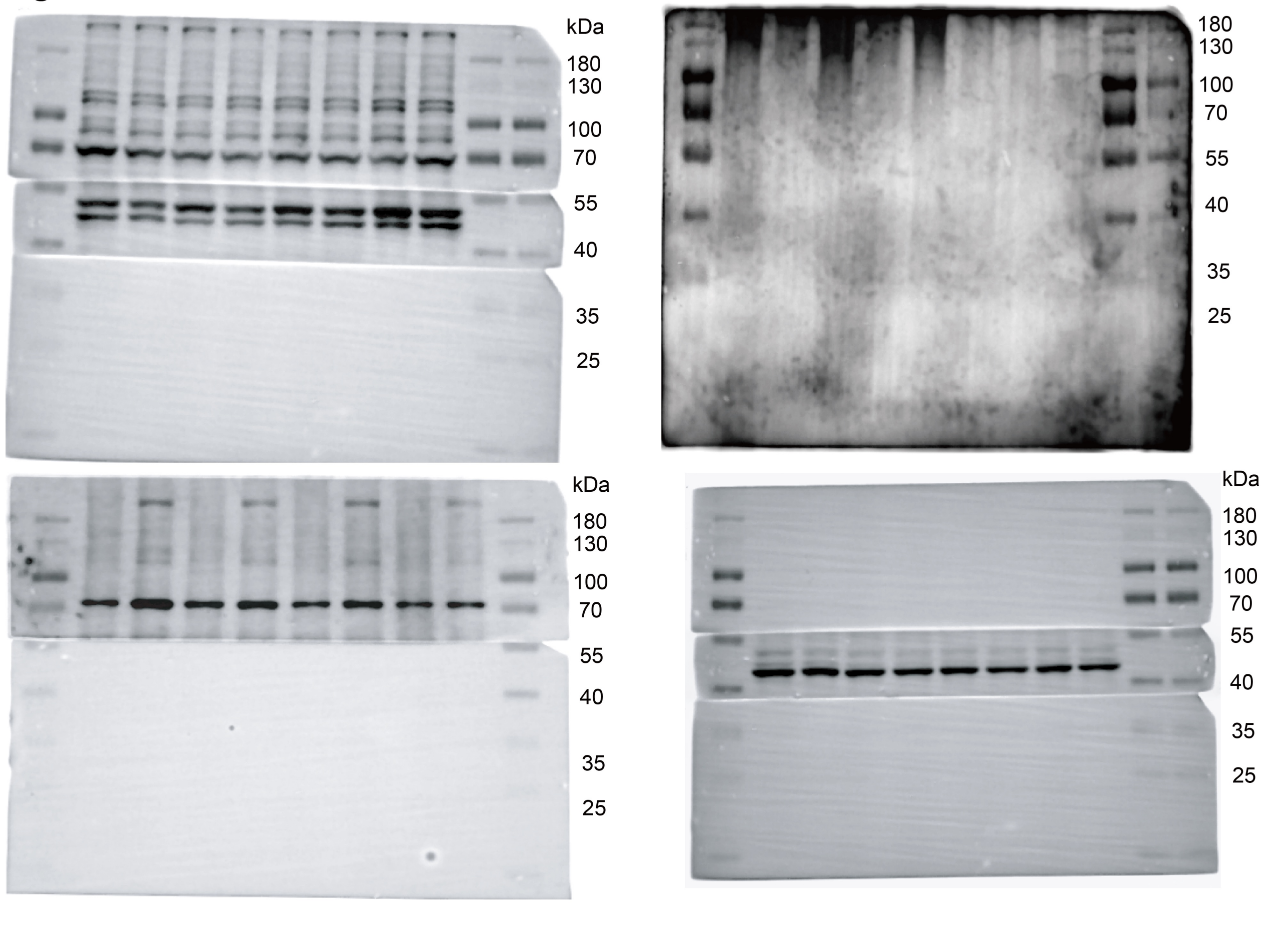

Figure S5A

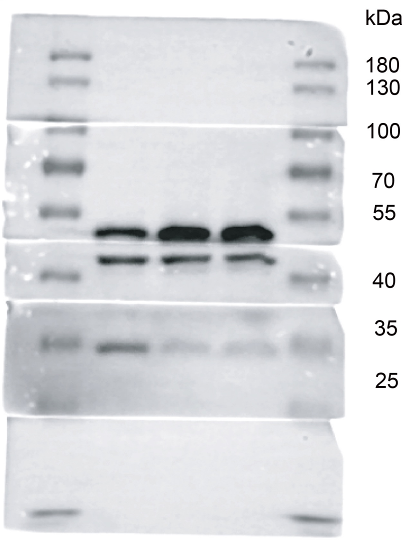

Figure S5B

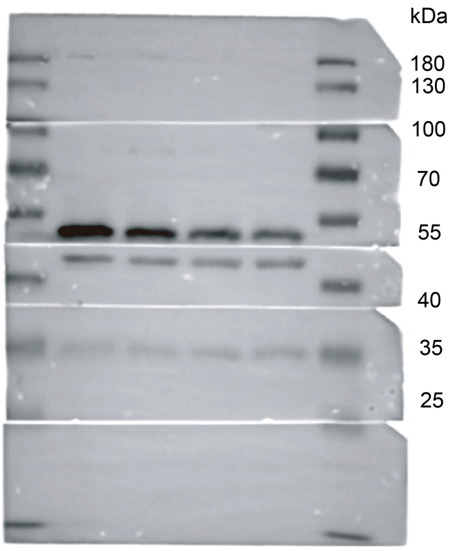

Figure S5C

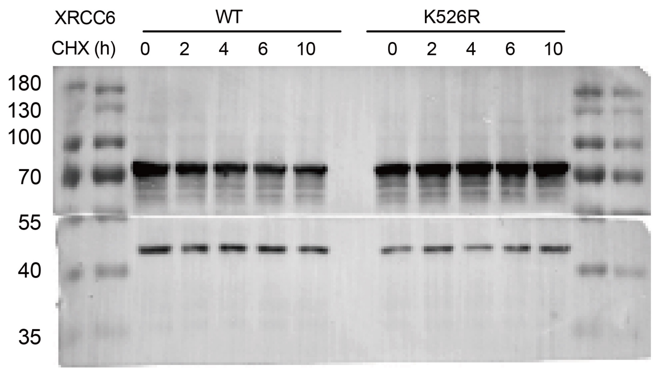

Figure S5D

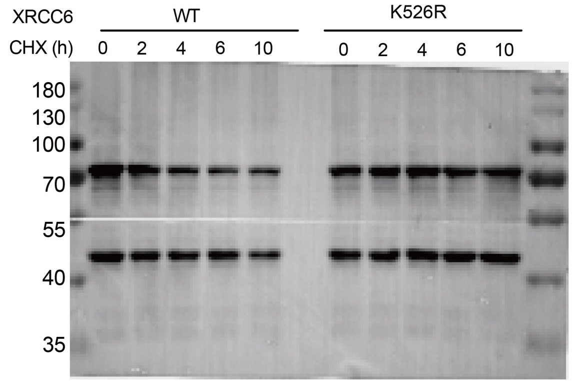

Figure S5E

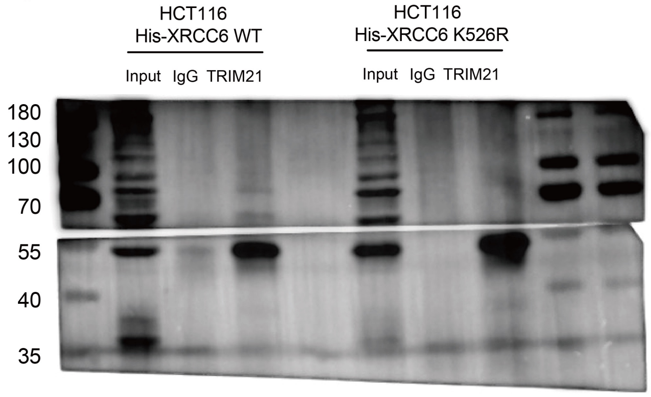

Supplement: Supplementary file 4 — WB Raw Data [file 41419_2025_8160_MOESM4_ESM.pdf]
